# Lifestyle Interventions to Prevent Type 2 Diabetes in Women with a History of Gestational Diabetes: A Systematic Review and Meta-Analysis through the Lens of Health Equity

**DOI:** 10.3390/nu15214666

**Published:** 2023-11-03

**Authors:** Gebresilasea Gendisha Ukke, Jacqueline A. Boyle, Ahmed Reja, Wai Kit Lee, Mingling Chen, Michelle Shi Min Ko, Chelsea Alycia, Jane Kwon, Siew Lim

**Affiliations:** 1 Health Systems and Equity, Eastern Health Clinical School, Monash University, Level 2, 5 Arnold Street, Box Hill, VIC 3128, Australia; gebresilasea.ukke@monash.edu (G.G.U.); jacqueline.boyle@monash.edu (J.A.B.); 2 School of Medicine, College of Health Sciences, Addis Ababa University, Addis Ababa 9086, Ethiopia; ahmedreja@yahoo.com; 3 Faculty of Medicine, Nursing and Health Sciences, Monash University, 264 Ferntree Gully Rd, Notting Hill, VIC 3168, Australia; wlee0019@student.monash.edu (W.K.L.); chelseaalycia18@gmail.com (C.A.); 4 Monash Centre for Health Research and Implementation, Monash University, Level 1, 43-51 Kanooka Grove, Clayton, VIC 3168, Australia; mingling.chen@monash.edu; 5 MD Programme, Duke-NUS Medical School, 8 College Rd, Singapore 169857, Singapore; michelle.ko.shi.min@u.duke.nus.edu; 6 Diabetes Victoria, Suite G01/15-31 Pelham St, Carlton, VIC 3053, Australia; jkwon@diabetesvic.org.au

**Keywords:** equity, diabetes gestational, lifestyle intervention, meta-analysis, progress, diabetes mellites type 2

## Abstract

Background: Type 2 diabetes mellites is one of the health problems disproportionally affecting people with low socioeconomic statuses. Gestational diabetes mellites increases the risk of type 2 diabetes by up to ten-fold for women. Lifestyle interventions prevent type 2 diabetes in women with prior gestational diabetes. However, it is unknown if similar effectiveness can be expected for all population subgroups. Objective: This study aims to assess the prevention of type 2 diabetes in women with prior gestational diabetes using population characteristics according to the PROGRESS (place of residence, race/ethnicity/culture/language, occupation, gender/sex, religion, education, socioeconomic status, and social capital) criteria. Methods: MEDLINE, CINAHL, EMBASE, PubMed, PsycINFO, Web of Science, and EBM Reviews databases were searched for interventional studies of diet, physical activity, or behavioural interventions published up to 21 February 2023. Random effects subgroup meta-analysis was conducted to evaluate the association of population characteristics and intervention effects. Results: All studies were conducted in high-income countries or middle-income countries. Two-thirds of the studies reported on race/ethnicity and education level. Less than one-third reported on place (urban/rural), occupation, and socioeconomic status. None reported on religion or social capital. Studies from high-income countries (MD = −1.46; 95% CI: −2.27, −0.66, *I*^2^ = 70.46, *p* < 0.001) showed a greater reduction in bodyweight compared with the studies conducted in middle-income countries (MD = −0.11; 95% CI: −1.12, 0.89, *I*^2^ = 69.31, *p* < 0.001) (*p* for subgroup difference = 0.04). Conclusion: There are significant equity gaps in the evidence for the prevention of type 2 diabetes in women with prior gestational diabetes due to reports on population characteristics being poor. Interventions may be less effective in reducing bodyweight in women from middle-income countries compared to high-income countries. Collecting and analysing data related to equity is needed to understand the effect of lifestyle interventions on type 2 diabetes for different population subgroups.

## 1. Introduction

Diabetes mellites is one of the health problems confounded by significant demographic, socioeconomic, and geographic disparities [1,2]. The International Diabetes Federation reported a continued rise in the global prevalence of diabetes in 2021, with 1 in 10 adults having diabetes, more than 90% of which are type 2 diabetes mellites (T2DM), and more than three-quarters that live in low- and middle-income countries (LMICs) [2]. Within countries, the risk of having T2DM also varies according to the socioeconomic status, with individuals with a low level of education or income or with certain occupations, such as manufacturing workers and cleaners, having a greater risk of T2DM [3,4,5,6]. These sociodemographic disparities were also seen in the screening and the lifestyle interventions used for T2DM prevention. In the United States of America (USA), Asian Americans were 34% less likely to receive diabetes screening than non-Hispanic whites [7].

Gestational diabetes mellites (GDM), a well-established risk factor for T2DM, is also increasing globally, but with a considerable variation from region to region [8]. The estimated global standardised prevalence of GDM for 2021 was 14.0%, with the highest prevalence in the Middle East and North Africa (27.6%) and the lowest prevalence in North America (7.1%) [8]. The incidence of GDM also varies by ethnicity [9,10]. For example, in the USA, the greatest rate of increase in GDM was among Asian Indians [10]. Moreover, the incidence of T2DM after a patient acquires GDM has been reported to be higher in non-White women (15.6%) than White women (9.9%) [11].

Health equity is the absence of unfair and avoidable differences in health among population groups that are defined socially, economically, demographically, or geographically [12]. Health equity can be facilitated by ensuring that the needs of groups or persons most at risk for poor health are addressed. Among the factors that determine people’s health outcomes and health behaviours are the social determinants of health [1,13,14,15]. Social determinants of health are defined as the conditions in which people are born, grow up, live, work, and age (such as income, education, employment, and a broader set of forces and systems influencing the conditions of day-to-day life) [16]. Even though the concept of health equity has a long history, disparities in access to health services and health outcomes continue to occur among different populations [17,18]. Julian Tudor Hart described this disparity in medical care in 1971 as the inverse care law, which states that “the availability of good medical care tends to vary inversely with the need for it in the population served” [19]. This is also true for preventive health services, in which the inverse prevention law states that “those in most needs of benefiting from preventive interventions are least likely to receive them” [20]. This was illustrated through the diabetes prevention program in England, in which people living in the more deprived areas were under-represented [21]. Thus, inequitable preventive efforts could widen existing health inequalities.

One of the ways to understand health equity in research is to identify participant characteristics with social determinants of health according to the PROGRESS (place of residence, race/ethnicity/culture/language, occupation, gender/sex, religion, education, socioeconomic status, and social capital) framework [22]. The use of the PROGRESS framework ensures that socially stratifying factors are comprehensively considered when conducting, reporting, and assessing the effect of an intervention [23]. To date, the consideration of the social determinants of health in intervention studies aiming at preventing T2DM among women with a history of GDM has not been systematically studied. Therefore, this study aimed to assess T2DM prevention research in women with a history of GDM according to the PROGRESS criteria.

## 2. Materials and Methods

### 2.1. Search Strategy and Selection Criteria

Following the Preferred Reporting Items for Systematic Reviews and Meta-Analyses (PRISMA) guidelines [24], we systematically searched published articles with MEDLINE, CINAHL, EMBASE, PubMed, PsycINFO, Web of Science, and EBM Reviews published up to 21 February 2023. The search strategy for MEDLINE is available in Supplementary Table S1. We screened the reference lists of reviewed articles for potentially relevant articles that the electronic search might have missed. We searched the International Clinical Trial Registry Platform. We did not limit our search by language. We registered the protocol with PROSPERO (CRD42022314231).

We included randomised controlled trials, non-randomised controlled trials, and pre–post single-arm studies that tested the effect of physical activity, diet, behavioural interventions, or a combination of these interventions. We included studies conducted among women with a history of GDM during postpartum or those that started prenatally and continued postpartum. We did not limit our search by postpartum age. We included studies that reported at least one of the following glycaemic, anthropometric, or behavioural outcomes: bodyweight, body mass index, waist circumference, T2DM, 2 h postprandial glucose, fasting blood glucose, haemoglobin A1c, homeostatic model assessment for insulin resistance, physical activity, fibre intake, fat intake, and total energy intake. Studies that we excluded were those that involved women with type 1 diabetes or T2DM, pharmacological or supplementation interventions, prenatal-only interventions that did not continue after birth, or those that combined pharmacological or supplementation interventions with lifestyle interventions. We excluded conference abstracts, letters, editorials, narrative reviews, systematic reviews, study protocols, trial registries, commentaries, and dissertations. Two independent reviewers (G.G.U. and M.C. or J.K.) screened all of the articles for titles/abstracts and full text. Discrepancies were discussed, and the consensus was achieved or resolved with an arbitrator (S.L.).

### 2.2. Quality Assessment

We used the revised Cochrane risk of bias tool for randomised trials to assess the risk of the included randomised controlled trials [25]. This tool assesses the risk of bias using five domains (one additional domain for cluster randomised controlled trials) and rates the risk as low, with some concerns, and high. For the non-randomised controlled trials, we used the Risk of Bias in Non-randomised Studies of Interventions tool [26]. This tool assesses the risk of bias using seven domains and rates the risk as low, moderate, or critical. For the quality assessment of the single-arm studies, we used the Newcastle-Ottawa Scale which assesses the quality of the studies using three main domains [27]. We rated the quality of the single-arm studies as good quality: 7–9 points, fair quality: 5–6 points, and poor quality: 0–4 points. Two reviewers (G.G.U. and M.C. or M.S.M.K.) independently assessed the quality of the included studies. We resolved conflicts through discussion.

### 2.3. Data Extraction

G.G.U. and W.L. or C.A. independently extracted the data from the included studies. Extracted data included the following: author and publication year, study country, study’s funder, study setting, study design, time of intervention, sample size, baseline characteristics of participants, and study outcomes. When reported, we extracted the data according to the PROGRESS checklist on race/ethnicity/culture/language, occupation, gender/sex, religion, education, socioeconomic status, and social capital. We contacted the corresponding authors for any missing data. Discrepancies were resolved through consensus (G.G.U. and W.L. or C.A.) or with an arbitrator (S.L.).

### 2.4. Data Synthesis and Analysis

The primary focus of this analysis comprises the PROGRESS characteristics of the included studies and the effects of a lifestyle intervention on T2DM, bodyweight, and body mass index using PROGRESS characteristics. We categorised the place of residence of the study participants based on the country where the study was conducted. Classification of the country was conducted according to the World Bank as high income, middle income (upper and lower), or low income, the continent in which the country is located, and if it is self-reported as an urban or rural [28]. For race or ethnicity, the data were extracted as reported by the individual papers, including country of birth (e.g., Australian) [29], country of origin (e.g., Mexican Americans) [30,31], region of birth (e.g., South Asian) [32], or cultural background based on the geographic region (e.g., Asian) [33]. If eighty percent or more of the participants were from a specific ethnic group in a particular study, as reported by the authors, the study was deemed to have been conducted among that particular ethnic group; if the majority ethnicity group comprised less than 80% of all participants, the study was considered as conducted in a mixed population [11,34]. If the population of the country in which the study was conducted was homogeneous (with more than 80% of participants being from a particular ethnic background (e.g., Tianjin, China) [35]) and ethnicity was not reported, all participants were considered to be from the majority ethnicity in the country [11]. For occupation/employment, we classified the studies into two categories: studies in which more than 50% of the participants were in paid employment and those in which less than 50% were in paid employment, including homemakers. We categorised the educational status of the participants into two groups: studies in which 50% or more of the participants had tertiary education and those in which less than 50% attained tertiary education. Here, tertiary education level refers to any study after high school, including college undergraduate degrees, postgraduate degrees, vocational schools, and non-degree certificate programs described in different ways in the included studies. For the socioeconomic status, as the average monthly income is not the same for all countries, we used the average income of the individuals in the country where the study was conducted in the same year as a cutoff point. Then, we categorised the studies into studies in which more than, or equal to, half of the study participants had a monthly income of more than, or equal to, the average value for that country and less than the average value.

We performed meta-analyses of randomised controlled trials and non-randomised controlled trials with subgroup analyses to investigate the effects of these social determinates of health on a lifestyle intervention using PROGRESS characteristics of the following primary outcomes: T2DM, bodyweight, and body mass index. For the incidence of T2DM, we calculated the risk ratio with a 95 percent confidence interval (CI) using the random effects model and the DerSimonian and Laird method. For continuous outcomes, we calculated mean differences (MD) with a 95% CI. The *I*^2^ test determined statistical heterogeneity. We considered *I*^2^ values of more than 50% to be moderate to high heterogeneity [36]. *p* < 0.05 was considered statistically significant. We used funnel plots and the Egger test to display the publication bias of the included studies. We used Stata statistical software (Release 16; College Station, TX, USA; StataCorp LLC) for the statistical analyses [37].

## 3. Results

**Figure 1:**
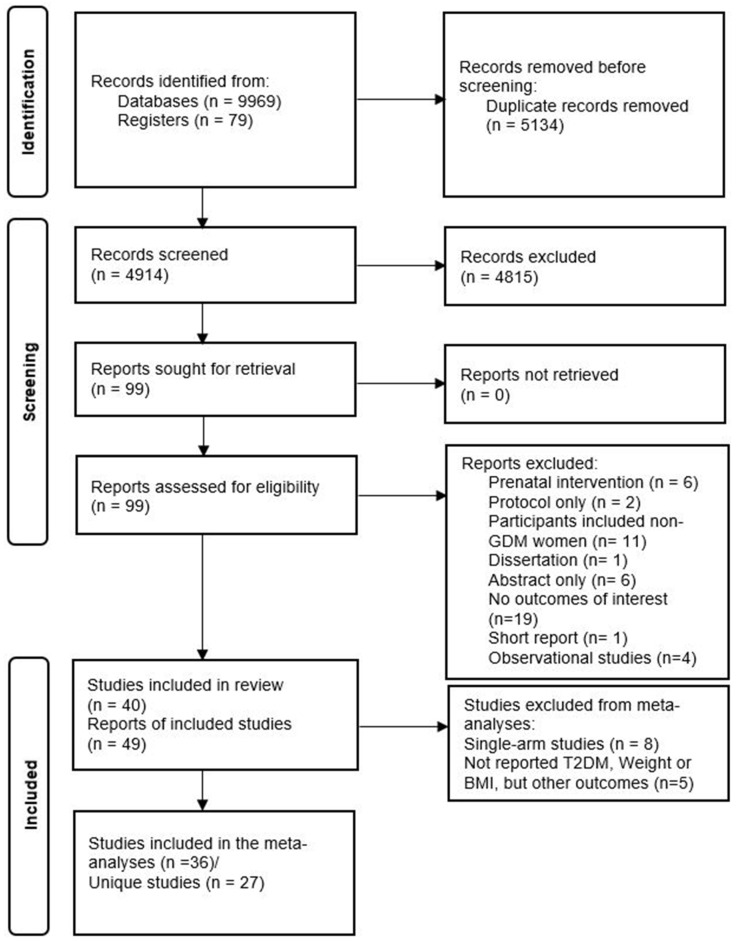
PRISMA diagram of included studies.

Our search identified 10,048 records overall, and 4914 records without duplicates, of which 99 were eligible for full-text review. Fifty records were excluded during the full-text review. Reasons for exclusion are depicted in Figure 1. Finally, 49 records (40 unique studies) were included in this systematic review. This meta-analysis included 26 unique studies that reported the primary outcomes using the PROGRESS criteria (Figure 1).

### 3.1. Risk of Bias and Quality Assessment

Of the included studies, 20 (69.0%) of the randomised controlled trials had a high risk of bias, mainly due to a deviation from the intended intervention, 4 (13.8%) with some concerns, and 5 (17.2%) with a low risk of bias. Of the eight single-arm studies, only one had a good quality, four had fair quality, and three had poor quality. Out of the two non-randomised controlled trials, one had a serious risk of bias and the other a moderate risk of bias (Table S2).

### 3.2. Study Characteristics of PROGRESS Framework

#### 3.2.1. Place of Residence (P)

The studies were mostly conducted in three countries, as follows: 25.0% were from Australia, 22.5% were from the USA, and 17.5% were from China. The rest (35.0%) were from 11 different countries. Overall, 72.5% of the studies were from high-income countries (HIC), 22.5% were from upper/middle-income countries, and 5.0% were from lower/middle-income countries, with no studies from low-income countries. Only 22.5% reported the location of residence of the participants, which were classified as urban, rural, or semi-urban. Of these, 55.6% of the studies were conducted in urban/semi-urban areas or included urban or semi-urban women, [35,38,39,40,41] 11.1% included both urban and rural women, [42] and 33.3% included rural women [40,43,44,45,46]. None of the studies reported on other specific examples of places of residence which were associated with disadvantages, such as slum areas (Table 1).

#### 3.2.2. Race/Ethnicity/Culture/Language (R)

Out of the 40 studies included in this review, only 27 (67.5%) studies reported on the ethnicity of their participants. Most of these studies (45.7%) included women from a range of mixed ethnic backgrounds, 22.9% were conducted among East Asians (mainly Chinese), 17.1% among Whites, 2.9% among Middle Easterners, 5.7% among South Asians, and 5.7% among Mexican Americans. Only two studies aggregated the effect of a lifestyle intervention with ethnicity [32,47]. Ten (25%) studies excluded women who had difficulties in communicating in English or other primary languages in the study area [33,48,49,50,51,52,53,54,55,56] (Table 1).

#### 3.2.3. Occupation (O)

The employment status of the participants was reported only by a quarter of the studies (Table 1). Among the studies that reported employment, half of them mainly included participants that were employed. However, none of the studies reported on the type of occupation of the study participants. 

#### 3.2.4. Gender (G)

All the studies were conducted in women with a history of GDM. There was no other information on gender.

#### 3.2.5. Religion (R)

None of the studies reported the religion of the participants. 

#### 3.2.6. Education (E)

The education level of the participants was reported by 57.5% of the studies. Out of the studies that reported education, 73.9% studies mostly included participants with a tertiary level of education (Table 1 and Table 2).

#### 3.2.7. Socioeconomic Status (S): Income

Less than one-third of the studies reported the income of participants. Out of the studies that reported income, more than 61% included participants with a monthly income above the average for the country where the study was conducted. Only one study mentioned how the study planned to include participants of different socioeconomic status [43] (Table 1). 

#### 3.2.8. Social Capital (S)

None of the studies reported the social capital of the participants.

**Table 1:** Summary of the included studies according to intervention type and PROGRESS ^a^ characteristics.

Study	Sample	Country Classification	Intervention Type (Diet or Physical Activity)	Country	Residence (Urban vs. Rural)	Ethnicity	Occupation	Educational Status	Income Level ^c^
Brazeau 2014 [57]	36	HIC	Combined	Canada	NR	NR; not specified	NR	NR	NR
			Combined						
Brokaw 2018 [42]	283	HIC	Combined	USA	Urban and rural	NR; not specified	NR	NR	NR
Cheung 2011 [58]	43	HIC	Physical activity	Australia	NR	NR; not specified	NR	NR	NR
Cheung 2019 [32]/2022 [59]	60	HIC	Combined	Australia	NR	Mixed: South Asian, Southeast Asian, Australian, others	NR	NR	NR
Ferrara 2011 [48]	197	HIC	Combined	USA	NR	Mixed: Non-Hispanic white, Black/African American, Asian or Pacific Islander, Hispanic origin, others	≥50% employed	≥50% tertiary	NR
Ferrara 2016 [60]	2280	HIC	Combined	USA	NR	Mixed: Asian, Non-Hispanic white, Hispanic, African American, multiracial, Pacific Islander, others	NR	NR	NR
Geng 2014 [49]	100	MIC	Combined	China	NR	East Asian (Chinese) ^b^	NR	NR	NR
Guo 2021 [43]; Chen 2022 [45]; Zhong 2023 [46]	320	MIC	Combined	China	Rural	East Asian (Chinese)—Han and others	≥50% employed	<50% tertiary	High
Holmes 2018 [50]	60	HIC	Combined	UK (Ireland)	NR	White	≥50% employed	≥50% tertiary	NR
Hu 2012 [35]; Liu 2008 [61]	1180	MIC	Combined	China	Urban	East Asian (Chinese) ^b^	NR	≥50% tertiary	Low
Kapoor 2019 [62]	56	MIC	Combined	India	NR	South Asian: Indian	<50% employed	≥50% tertiary	NR
Kim 2012 [63]	49	HIC	Physical activity	USA	NR	Mixed: Non-Hispanic white, Asian (South and East), African American, others	NR	≥50% tertiary	High
Kim 2021 [64]	119	HIC	Combined	South Korea	NR	East Asian (Korean) ^b^	NR	NR	NR
Lee 2022 [65]	298	MIC	Combined	Malaysia	Urban and semi-urban	Mixed (mixed Asians): Malays, Chinese, Indians, others	NR	<50% tertiary	NR
Li 2021 [40]	404	MIC	Combined	China	Rural	East Asian (Chinese): Han and others	<50% employed	<50% tertiary	High
Lim 2017 [39]	33	HIC	Combined	Australia	Urban	Mixed: Australia-born and born outside Australia	NR	≥50% tertiary	High
Lim 2021 [41]	200	HIC	Combined	Singapore	Urban	Mixed (Mixed Asians): Malays, Chinese, Indians, others	≥50% employed	≥50% tertiary	NR
Man 2021 [47]; Aroda 2015 [66]; Ratner 2008 [67]	350	HIC	Combined	USA	NR	Mixed: White, African American, Hispanic, others	NR	NR	NR
McCurley 2017 [30]	24	HIC	Combined	USA	NR	Mexican Americans	NR	<50% tertiary	Low
McIntyre 2012 [68]	28	HIC	Combined	Australia	NR	NR; not specified	NR	≥50% tertiary	NR
McManus 2018 [51]; Barton 2019 [69]	178	HIC	Combined	Canada	NR	Mixed: White and others	NR	≥50% tertiary	High
Nicholson 2016 [70]	23	HIC	Combined	USA	NR	Mixed: White, African American, Asian, Hispanic, others	≥50% employed	≥50% tertiary	NR
Nicklas 2014 [52]	75	HIC	Combined	USA	NR	Mixed: White, African American, Asian, Hispanic or Latina	NR	≥50% tertiary	High
O’Dea 2015 [54]	50	HIC	Combined	Ireland	NR	White ^b^	NR	NR	NR
O’Reilly 2016/2019 [33,71]	573	HIC	Combined	Australia	NR	Mixed: Asian, Australian and New Zealander, Aboriginal, and Torres Strait Islander	<50% employed	≥50% tertiary	High
Peacock 2015 [53]	31	HIC	Combined	Australia	NR	White	NR	NR	NR
Perez-Ferre 2015 [72]	237	HIC	Combined	Spain	NR	Mixed: White and Hispanic	NR	NR	NR
Philis-Tsimikas 2014 [31]	84	HIC	Combined	USA	NR	Mexican Americans	<50% employed	<50% tertiary	Low
Potzel 2022 [56]	66	HIC	Combined	Germany	NR	White ^b^	NR	≥50% tertiary	NR
Rautio 2014 [73]	115	HIC	Combined	Finland	NR	White ^b^	NR	NR	NR
Reinhardt 2012 [44]	38	HIC	Combined	Australia	Rural	NR; not specified	NR	NR	NR
Rollo 2020 [29]	29	HIC	Combined	Australia	NR	White	NR	≥50% tertiary	High
Shek 2014 [74]	450	MIC	Combined	China	NR	East Asian (Chinese)	NR	NR	NR
Sheng Yu 2012 [75]	130	MIC	Combined	China	NR	East Asian (Chinese) ^b^	NR	≥50% tertiary	Low
Shyam 2013/15 [76,77]	77	MIC	Combined	Malaysia	NR	Mixed (Mixed Asians): Malays, Chinese, Indians, others	NR	≥50% tertiary	Low
Smith 2014 [55]	59	HIC	Combined	Australia	NR	Mixed: Australian, Asian	NR	NR	NR
Tandon 2022 [38]	1612	MIC	Combined	Bangladesh India Sri Lanka	Urban	South Asian: Bengali Indian, Singhalese	<50% employed	≥50% tertiary	NR
Wein 1999 [78]	200	HIC	Diet only	Australia	NR	Mixed: Australian and New Zealander, Mediterranean and Middle Eastern, Northern European, Southeast Asian, Indian subcontinental	NR	NR	NR
Yu Xiao 2012 [79]	126	MIC	Combined	China	NR	East Asian (Chinese) ^b^	NR	NR	NR
Zilberman-Kravits 2018 [80]	180	HIC	Combined	Israel	NR	Middle Eastern (Jewish and Bedouins)	NR	<50% tertiary	NR

Note: No study reported religion or social capital. HIC: high-income country; MIC: middle-income country; NR: not reported; ^a^ PROGRESS: place of residence, race/ethnicity/culture/language, occupation, gender/sex, religion, education, socioeconomic status, and social capital. ^b^ Ethnicity was not reported by the authors. However, it was determined based on the predominate ethnicity of the country. ^c^ High = when more than, or equal to, 50% of the participants’ income is above the average income for the country’s population where the study was conducted, during the same year. Low = when less than 50% of the participants’ income is above the average income for the country’s population where the study was conducted, during the same year.

**Table 2:** Summary of the included studies of PROGRESS characteristics.

PROGRESS Characteristics	Number Studies	Number of Participants
Place of residence country based on economy (World Bank Classification)		
High-income country	29	5700
Upper/middle-income country	9	3085
Lower/middle-income country	2	1668
Low-income country	0	0
Continent		
Asia	14	5252
Australia	10	1094
North America	11	3579
Europe	5	528
South America	0	0
Africa	0	0
Asia (*n* = 14)		
China	7	2710
Malaysia	2	375
Singapore	1	200
India, Sri Lanka, and Bangladesh	1	1612
South Korea	1	119
India	1	56
Israel (Near East)	1	180
North America (*n* = 11)		
USA	9	3365
Canada	2	214
Europe (*n* = 4)		
Ireland (UK)	2	110
Spain	1	237
Finland	1	115
Germany	1	66
Urban vs. Rural		
Urban	4	3025
Urban and semi-urban	1	298
Urban and Rural	1	283
Rural	3	762
NR	31	6085
Ethnicity ^a^		
Mixed ^b^	16	4889
East Asian	8	2829
White	6	351
South Asian	2	1668
Mexican Americans	2	108
Middle Eastern	1	180
Not specified ^c^	5	428
Occupation/employment		
Reported	10	3529
Not reported	30	6924
Occupation reported (*n* = 10)		
Mostly unemployed	5	2729
Mostly employed	5	800
Gender: women	40	10,457
Religion: not reported	40	10,457
Educational status	Number studies	Participants
Reported	23	58,876
Not reported	17	4577
Educational status reported (*n* = 23)	Number studies	Participants
Mostly with tertiary education	17	4566
Mostly without tertiary education	6	1310
Socioeconomic status/income (*n*= 39)	Number studies	Participants
Reported	13	3156
Not reported	27	7297
Income reported (*n* = 13)	Number studies	Participants
Above average	8	1661
Below average	5	1495
Social capital not reported	40	10,457

^a^ Ethnicity for eight studies was not reported by the authors and was determined based on the predominant ethnicity of the country. ^b^ Six out of the sixteen studies with mixed ethnicity were from the USA, five were from Australia, two were from Malaysia (Mixed Asian), one was from Singapore (Mixed Asian), one was from Canada, and one was from Spain. The authors reported the following ethnicities: Asian, Middle Eastern, non-Hispanic white, Hispanic, African American, and Pacific Islander. ^c^ The authors did not report on ethnicity; the studies were from multiethnic countries. Therefore, ethnicity could not be determined.

### 3.3. Meta-Analysis

#### Intervention Effect of PROGRESS Characteristics

**Figure 2:**
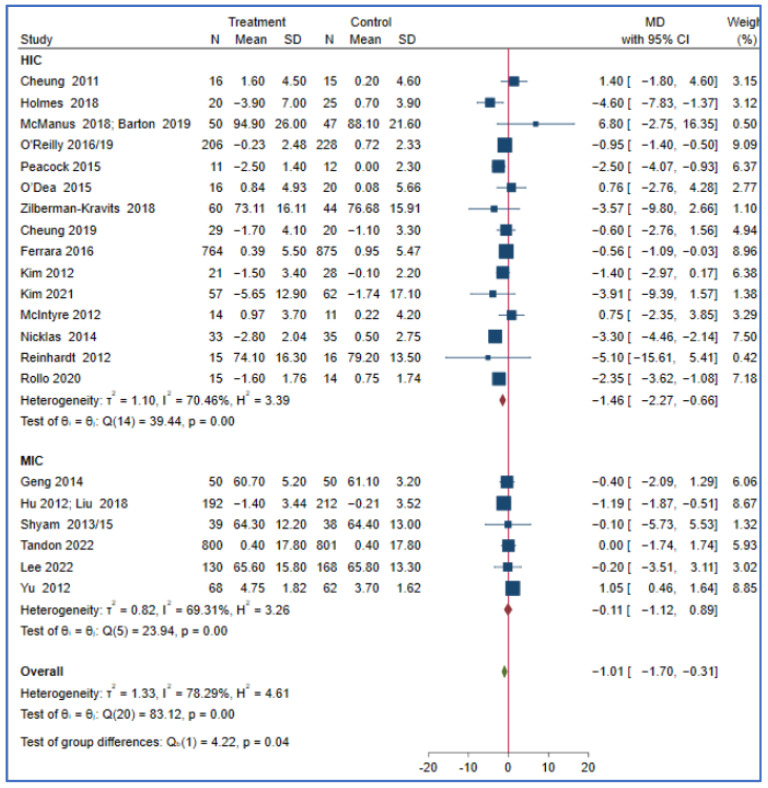
The effect of a lifestyle intervention on bodyweight (by country) according to the World Bank classification (HIC: high-income countries, MIC: middle-income countries) [29,32,33,35,38,44,49,50,51,52,53,54,58,60,61,63,64,65,68,69,71,75,76,77,80].

This meta-analysis showed that the effect of a lifestyle intervention on T2DM and body mass index did not differ in the reported PROGRESS characteristics, such as ethnicity, income, education, and occupation. Studies from HICs may have a greater reduction in bodyweight (MD = −1.46; 95% CI: −2.27, −0.66, *I*^2^ = 70.46, *p* < 0.001) compared to those conducted in MICs (MD = −0.11; 95% CI: −1.12, 0.89, *I*^2^ = 69.31, *p* < 0.001) (P for subgroup difference = 0.04). The effect of a lifestyle intervention on bodyweight did not differ in other reported PROGRESS characteristics, including the sensitivity analyses, as the studies that reported the variables are few (Figure 2 and Table S3).

## 4. Discussion

We evaluated T2DM prevention for women with a history of GDM with population characteristics using the PROGRESS criteria. This review highlights both a lack of reporting that enables assessment of equity and, in those where some equity characteristics could be assessed, a lack of the inclusion of participants at the highest risk of T2DM. Most studies did not report on one or more of the PROGRESS criteria, with the most frequently reported criteria being ethnicity (67.5%) and education (55.7%). Only one-quarter of the studies reported on the urban or rural locality of residence, one-third on the income level, and none on the social capital or religion of their participants. All studies were conducted in HICs or MICs (mostly upper middle) and, of those that did report findings based on the PROGRESS criteria, participants were mostly (73.9%) tertiary educated and had a high level of income (61.5%). A lifestyle intervention was effective in reducing bodyweight in studies from HICs in comparison with MICs. Furthermore, other PROGRESS characteristics showed no difference in the effect of a lifestyle intervention on T2DM, bodyweight, or body mass index.

This review highlights the mismatch in the research in T2DM prevention and the prevalence of T2DM (by geographic region). There were no studies in low-income countries in Africa or the Pacific region despite these regions being disproportionately burdened with T2DM and GDM [8,81]. The greater representation of HIC in these studies suggests that the current research studies are not targeting areas with a higher burden of T2DM and GDM, where the burden of the problem is rising, or where current ‘evidence-based’ interventions might not be culturally appropriate for the prevention of T2DM in women with a history of GDM outside HICs [1,2,8].

We also found a differential intervention effectiveness by region, with T2DM prevention interventions for women with prior GDM being more effective in reducing bodyweight (a key factor in reducing the incidence of T2DM) [82] in HICs compared with MICs. The greater effectiveness in weight reduction in HICs may be attributed to the difference in the intervention delivery mode: more than half of the studies from HICs were delivered virtually, and another one-third had at least one virtual component, while no studies from MICs were delivered virtually only. This meta-analysis also showed that virtually delivered interventions have a better effect than those with both virtual and in-person components, with in-person delivered interventions being the least effective in women with a history of GDM. After childbirth, women are often busy with childcare-related duties. Attending interventions delivered in person at health centres or hospitals may be challenging; thus, virtual interventions may be easier to access [83,84]. However, the virtual mode requires internet access and digital and language literacy [85]. Of the virtually delivered interventions from HICs, those that reported the educational status of participants identified that most of them had tertiary level education [29,52,63,68]. Results reporting success may not be applicable to other women in HICs where access may be an issue, such as those from low socioeconomic status groups, women of migrant background, or Indigenous peoples (where program adaptation may be required) [86,87]. Virtual program delivery may not be accessible to many women from LMICs, particularly those from rural areas [29,52,87,88]. Adopting the virtual mode of delivery without considering the needs of women without internet access and their literacy could lead to the fulfilment of the inverse prevention law [20].

There was also a mismatch between research effort and disease burden in relation to urban/rural regions within countries. There is a higher burden of T2DM in rural areas, including in HICs such as the USA and Australia [89,90]. However, studies infrequently documented the residential location or the inclusion of women from non-urban locations. As T2DM is also increasing outside major cities due to changes in lifestyles and rises in obesity [91,92], it is important to understand the effectiveness of interventions in the rural context [93]. Some evidence show that lifestyle interventions can improve behavioural outcomes and psychological domains among rural women with a history of GDM [40,43,44].

There are ethnic disparities in the overall prevalence of T2DM as well as in the progression of GDM into T2DM [9,10,11,15], and an adequate representation of ethnic groups bearing the greater burden of the problem and the disaggregation of data, where feasible, is needed in the research to better understand the effectiveness of interventions in these groups. However, one-third of the studies included in this review did not report on ethnicity. Moreover, the authors reported ethnicity in various ways, and some used broad ethnic categories, such as Asian [33] or South Asian [32], making it challenging to understand the effect of lifestyle interventions based on disaggregated ethnic groups [94]. This meta-analysis of lifestyle interventions in the prevention of T2DM in the general population showed significant subgroup differences (by ethnicity) for some of the predictors of T2DM, such as 2 h glucose, weight, body mass index, waist circumference, and HbA1c [34,95]. Given the ethnic disparities in the prevalence of T2DM and the differential effectiveness of T2DM prevention (by ethnicity) in the general population, disaggregated data is needed to determine the effectiveness of lifestyle interventions in preventing T2DM in women a given ethnicity or racial background prior to GDM.

Our review also found that most studies did not report social determinants of health, such as education, income, and occupation [93]. Among those that reported these social determinants, results indicated that studies tended to include participants who were more educated or had higher incomes. However, those most in need of effective interventions are those with lower incomes or less education—in whom there is a higher and rising burden of T2DM and GDM [3,4,6]. In the general population at risk of cardiovascular or T2DM risk, lifestyle interventions conducted in low-income individuals or those with food insecurity usually show promising results post-intervention, although benefits gradually decrease over time [96,97]. Women with a history of GDM and low income may also show the same trend, which may necessitate additional means to sustain the effect of the intervention for a more extended period of time. A systematic review of determinants of adherence to lifestyle interventions in adults with obesity also identified that socioeconomic constraints and a lack of knowledge are among the barriers to behavioural change [98]. When proving aid to women from low-income countries, giving more attention to behavioural change techniques, such as goal setting and being aware of costs and literacy barriers, is also needed [99]. Failing to consider groups that experience structural disadvantages may also lead to the fulfilment of the inverse prevention law, in which the least preventative measures occur among those who need it most [20].

None of the studies reported the religion of the participants, which could be due to religious affiliation being perceived as an ethically and politically sensitive characteristic in many parts of the world [100,101]. Our findings are in line with a past study investigating equity in people that were vaccinated, in which religion was one of the least reported equity attributes [102]. As none of the studies in this review reported religion, it is not possible to know if there was any disparity regarding it. Religion has been reported as both a barrier and an enabler of health outcomes [103,104], and discrimination by religion has been reported as one of the barriers to healthcare [105]. Providing culturally/religiously competent interventions can improve the outcomes of lifestyle interventions in the same way as in patient care [104]. Being associated with a religious organisation or community is also related to social capital, which can be a support for positive health behaviours. Social capital is an intangible but important social resource which can reduce immediate barriers to the access to healthcare for non-communicable disease prevention; however, it was not reported by any of the studies in this review [106]. Social capital has been shown to be a protective measure against chronic diseases, such as diabetes [107]. It has significant practical value for patients with diabetes as it improves their quality of life and hence can provide inspiration and enlightenment for the prevention of diabetes [108,109]. Given its considerable benefits, the research gap implies that there is a need to consider participants’ social capital in the future by adopting the World Bank’s Integrated Questionnaire for Measuring Social Capital [110].

## 5. Strengths and Limitations

To the best of our knowledge, this systematic review and meta-analysis is the first to examine the effect of lifestyle interventions on T2DM prevention in women with a history of GDM based on population characteristics using the PROGRESS criteria as the framework. This review is novel in identifying a significant inequity in diabetes prevention research in women with a history of GDM. However, as most PROGRESS elements (such as income, occupation, and educational status) were reported differently, these were coded and categorised by the reviewers. Moreover, some PROGRESS characteristics (such as place (urban/rural), occupation, income, religion, and social capital) were rarely reported (if at all); thus, this limited the ability of this review to evaluate these population characteristics. Furthermore, substantial heterogeneity between studies needs to be considered when interpreting the results of this meta-analysis. Finally, the high risk of bias in most studies, which contributes to the main reason for the low certainty of evidence, also needs to be considered when applying the results of this meta-analysis.

## 6. Conclusions

There is considerable inequity in the research of T2DMprevention in women with a history of GDM. Nearly all of the studies were conducted in HICs or upper MICs, with no studies in low-income countries. Most social determinants of health were not reported in the studies. When reported, most studies included participants with a higher education or income level. The only difference noted by equity was that lifestyle interventions may have a greater reduction in bodyweight in studies conducted in HICs compared to those conducted in MICs. To advance the understanding of T2DM prevention in all population subgroups, future researchers and funders need to close the equity research gap in the prevention of T2DM in women with a history of GDM by performing the following tasks: by focusing on the inclusion of disadvantaged groups (or groups which are under-represented) and by collecting and reporting disaggregated data on equity.

## Data Availability

The protocol was registered in the International Prospective Register of Systematic Reviews (PROSPERO): registration ID CRD42022314231.

## Supplementary Materials

The following supporting information can be downloaded at: https://www.mdpi.com/article/10.3390/nu15214666/s1, Table S1: Search strategy, Table S2: Risk of bias assessment, Table S3: Subgroup analyses of the effect of lifestyle intervention in women with a history of gestational diabetes on incidence of T2DM by PROGRESS framework, Table S4: Subgroup analyses of the effect of lifestyle intervention in women with a history of gestational diabetes on bodyweight by PROGRESS framework, Table S5: Subgroup analyses of the effect of lifestyle intervention in women with a history of gestational diabetes on BMI by PROGRESS framework, Figure S1: Funnel plots for publication bias, Figure S2: Forest plots.
