# Supplementary material for: Lifestyle Interventions to Prevent Type 2 Diabetes in Women with a History of Gestational Diabetes: A Systematic Review and Meta-Analysis through the Lens of Health Equity
Source: Nutrients. 2023 Nov 3;15(21):4666. doi: 10.3390/nu15214666 (PMC10649749; doi:10.3390/nu15214666)
Supplement: Supplementary file 1 [file nutrients-15-04666-s001.zip › nutrients-2625942-supplementary.pdf]

Lifestyle Interventions to Prevent Type 2 Diabetes in Women with A History of Gestational Diabetes: A Systematic Review and Meta-Analysis Through the Lens of Health Equity

Gebresilasea Gendisha Ukke[1], Jacqueline A Boyle[1], Ahmed Reja[2], Wai Kit Lee[3], Mingling Chen[4], Michelle Shi Min Ko[5], Chelsea Alycia[3], Jane Kwon[6], and Siew Lim[1*]

[1] Health Systems and Equity, Eastern Health Clinical School, Monash University, Level 2, 5 Arnold Street Box Hill, Victoria, 3128, Australia; gebresilasea.ukke@monash.edu (G.U.); jacqueline.boyle@monash.edu (J.B.); siew.lim1@monash.edu (S. L.)

[2] School of Medicine, College of Health Sciences, Addis Ababa University, Addis Ababa, Ethiopia; ahmedreja@yahoo.com

[3] Faculty of Medicine, Nursing and Health Sciences, Monash University, 264 Ferntree Gully Rd, Notting Hill Victoria, 3168, Australia; wlee0019@student.monash.edu (W.L.); caly0001@student.monash.edu /chelseaalycia18@gmail.com

[4] Monash Centre for Health Research and Implementation, Monash University, Level 1, 43-51 Kanooka Grove Clayton Victoria, 3168, Australia; mingling.Chen@monash.edu

[5] MD Programme, Duke-NUS Medical School, 8 College Rd, Singapore 169857; michelle.ko.shi.min@u.duke.nus.edu

[6] Diabetes Victoria, Suite G01/15-31 Pelham St, Carlton Victoria 3053, Australia; jkwon@diabetesvic.org.au

* Correspondence: siew.lim1@monash.edu (S. L.)

Outline:

Table S1. Search strategy

Table S2. Risk of bias assessment

Table S3. Subgroup analyses of the effect of lifestyle intervention in women with a history of gestational diabetes on incidence of T2DM by PROGRESS[a] framework

Table S4. Subgroup analyses of the effect of lifestyle intervention in women with a history of gestational diabetes on bodyweight by PROGRESS[a] framework

Table S5. Subgroup analyses of the effect of lifestyle intervention in women with a history of gestational diabetes on BMI by PROGRESS[a] framework

Figure S1. Funnel plots for publication bias

Figure S2. Forest plots

Table S1. Search Strategy (MEDLINE)

#	**Query**
1	Diabetes, Gestational/
2	gestational diabetes.mp.
3	gestational diabetes mellitus.mp.
4	GDM.mp.
5	1 or 2 or 3 or 4
6	Diabetes Mellitus, Type 2/
7	Glucose Intolerance/
8	diabetes.mp.
9	diabetes mellitus.mp.
10	type 2 diabetes.mp.
11	type 2 diabetes mellitus.mp.
12	non-insulin dependent diabetes mellitus.mp.
13	NIDDM.mp.
14	impaired fasting glucose.mp.
15	IFG.mp.
16	Impaired glucose tolerance.mp.
17	IGT.mp.
18	maternal complication*.mp.
19	6 or 7 or 8 or 9 or 10 or 11 or 12 or 13 or 14 or 15 or 16 or 17 or 18
20	lifestyle.ab,ti.
21	behavio?r.ab,ti.
22	Diet*.ab,ti.
23	"diet reduce*".ab,ti.
24	"diet restricted*".ab,ti.
25	"nutriti*".ab,ti.
26	"educat*".ab,ti.
27	"exercis*".ab,ti.
28	"physical activit*".ab,ti.
29	20 or 21 or 22 or 23 or 24 or 25 or 26 or 27 or 28
30	"therap*".ab,ti.
31	"intervention*".ab,ti.
32	30 or 31
33	29 and 32
34	5 and 19 and 33

*= truncation

Table S2. Risk of bias assessment

a) Risk of bias of the included RCT studies

Study	Randomization process	Deviations from intended interventions	Missing outcome data	Measurement of the outcome	Selection of the reported result	Overall Bias
Cheung 2011[58]	Some concerns	High	High	Some concerns	Some concerns	High
Cheung 2019/2022 [32, 59]	Some concerns	High	Low	Low	Low	High
Ferrara 2011[48]	Some concerns	Low	High	Low	Low	High
Hu 2012;[35] Liu 2018[61]	Low	Low	Low	Low	Some concerns	Some concerns
Kim 2012[63]	Some concerns	Low	Low	Low	Low	Some concerns
Lim 2021[41]	Low	Low	Low	Low	Low	Low
McIntyre 2012[68]	Low	Low	Low	Low	Low	Low
McManus 2018;[51] Barton 2019[69]	Some concerns	High	Low	Low	Low	High
Nicklas 2014[52]	Low	High	Low	Low	Some concerns	High
O'Dea 2015[54]	Low	Low	High	Low	Low	High
O'Reilly 2916/2019[33, 71]	Low	High	Low	Low	Low	High
Peacock 2015[53]	Low	High	High	Low	Some concerns	High
Perze-Ferre 2015[72]	Some concerns	High	Low	Low	Some concerns	High
Ratner 2008;[67] Aroda 2015;[66] Man 2021[47]	Low	Low	Low	Low	Low	Low
Reinhardt 2012[44]	Some concerns	High	Low	Some concerns	Some concerns	High
Rollo 2020[29]	Some concerns	High	High	Low	Some concerns	High
Shek 2014[74]	High	High	Low	Low	Some concerns	High
Sheng 2012[75]	Some concerns	High	Low	Some concerns	Low	High
Shyams 2013/2015[76, 77]	Low	Low	Low	Low	Some concerns	Some concerns
Smith 2014[55]	Some concerns	Low	High	Low	Some concerns	High

Tandon 2022[38]	Low	Low	Low	Low	Low	Low
Wien 1999[78]	Some concerns	High	Low	Low	Some concerns	High
Yu Xiao 2012[79]	Some concerns	High	Low	Some concerns	Low	High
Zilberman K. 2018[80]	High	High	Low	Low	Some concerns	High

b) Risk of bias of the included cluster-RCT studies

Study	Randomization process	Identification or recruitment of participants	Deviations from intended interventions	Missing outcome data	Measurement of the outcome	Selection of the reported result	Overall Bias
Ferrara 2016[60]	Low	Low	Low	Low	Low	Low	Low
Guo 2021[43]; Chen 2022[45]; Zhong 2023[46]	Low	Low	Some concerns	Low	Low	Low	Some concerns
Holmes 2018[50]	Some concerns	Low	Low	High	Low	Some concerns	High
Lee 2022[65]	Low	Some concerns	High	High	Low	Low	High
Li 2021[40]	Low	Some concerns	Low	High	High	Low	High
Potzel 2022[56]	High	High	Low	Low	Some concerns	Some concerns	High

b) Risk of bias of the included non-RCT studies

Study	Confounding	Selection of participants	Classification of interventions	Deviations from intended interventions	Missing data	Measurement of outcomes	Selection of the reported result	Overall Bias
Kim 2021[64]	Low	Low	Low	Moderate	Moderate	Moderate	Low	Moderate
Geng 2014[49]	Serious	Moderate	Low	Moderate	Low	Low	Moderate	Serious

d) Quality assessment of the included single-arm studies

NOS criteria	Study: Kapoor 2018	McCurley 2017	Brazeau 2014	Brokaw 2017	Philis-Tsimikas 2014	Lim 2017	Nicholson 2016	Rautio 2014
**A. Selection (maximum of four stars)**								
1. Representativeness of the exposed cohort	*	*	*	*	*	*	*	*
2. Selection of the non-exposed cohort								
3. Ascertainment of exposure		*			*	*	*	
4. Demonstration that outcome of interest was not present at the start of study	*	*	*	*	*	*	*	*
B. **Comparability (maximum of two stars)**								
1. Comparability of the cohort on the basis of the design or analysis	*			*	*	*		
**C. Outcome (maximum of three stars)**								
1. Assessment of outcome					*	*	*	*
2. Was follow-up long enough for outcomes to occur	*	*		*	*		*	*
3. Adequacy of follow-up of cohorts	*			*	*	*	*	
Total (maximum of nine stars)	5	4	2	5	7	6	6	4
The overall quality	Fair	Poor	Poor	Fair	Good	Fair	Fair	Poor

Note: * = Number of stars—Good Quality: 7-8 stars/points; Fair Quality: 5-6 stars /points; Poor Quality: 0 to 4 stars/points

e) Summary of risk of bias in RCT studies

**Randomization process**	**Deviations from intended interventions**	**Missing outcome data**	**Measurement of the outcome**	**Selection of the reported result**	**Overall Bias**
Low= 14	Low = 13	Low = 20	Low = 24	Low = 16	Low =5
Some concerns = 13	Some concerns = 2	Some concerns = 0	Some concerns = 4	Some concerns = 13	Some concerns = 4
High = 3	High = 14	High = 9	High = 1	High = 0	High = 21

f) Summary of all risk of bias assessment results

Summary		
**RCT (Risk of bias)**	**Non-RCT (Risk of bias)**	**Single-arm studies (Quality)**
Low = 5	Moderate = 1	Poor = 3
Some concerns = 4	Serious = 1	Fair = 4
High = 21		Good =1
**Total=30**	**Total =2**	**Total = 8**

Table S3. Subgroup analyses of the effect of lifestyle intervention in women with a history of gestational diabetes on incidence of T2DM by PROGRESS[a] framework

Analyses	Studies	Participants	Relative risk (95% CI)	I^2	*p*-value for subgroup difference
**Country**					0.56
High-income	6	848	0.69 [0.49, 0.96]	10.4	
Middle-income	8	3220	0.79 [0.56, 1.11]	37.1	
**Continent**					0.22
Asia	8	3220	0.79 [0.56, 1.11]	37.1	
Australia	2	227	0.97 [0.62, 1.53]	0.0	
Europe	1	237	0.65 [0.31, 1.35]	NA	
North America	3	384	0.51 [0.33, 0.79]	0.0	
**Educational status[b]**					0.29
Mostly without tertiary education	1	332	0.17 [0.75, 1.85]	NA	
Mostly with tertiary education	6	2384	0.88 [0.66, 1.18]	0.0	
**Income level[c]**					0.34
Average or above:	2	172	0.17 [0.02, 1.39]	0.0	
Below average	3	611	0.261[0.13, 2.83]	0.0	

[a] PROGRESS (Place of Residence, Race/ethnicity/culture/language, Occupation, Gender/sex, Religion, Education, Socioeconomic status, and Social)

[b] Only seven studies reported educational status.

[c] Mean income level for the country during the same year in which the study was conducted was taken as a cut-off point; NA: Not applicable.

Note: Subgroup analysis for ethnicity, gender, religion and social capital could not be performed (Ethnicity: studies that report on ethnicity included a range of mixed ethnicity and were not mutually exclusive. Religion and social capital: not reported; Gender NA). No studies were from low-income countries, Africa, South America, or the Pacific.

Table S4. Subgroup analyses of the effect of lifestyle intervention in women with a history of gestational diabetes on bodyweight by PROGRESS[a] framework

Analyses	Studies	Participants	Mean difference, kg (95% CI)	I^2	p-value for subgroup difference
**Place of residence**					
**Country**					0.04
High-income	15	2779	−1.46 [−2.27, −0.66]	70.46	
Middle-income	6	2610	−0.11[−1.12, 0.89]	69.31	
Continent					0.49
Asia	8	2833	−0.31[−1.31, 0.69]	63.49	
Australia	7	622	−1.23[−2.17, −0.30]	49.90	
Europe	2	81	−1.97[−7.22, 3.28]	79.35	
North America	4	1853	−1.46[−3.17, 0.24]	82.87	
**Educational status[b]**					0.91
Mostly without tertiary education	2	402	−0.94[−3.86, 1.98]	0.0	
Mostly with tertiary education	11	2959	−1.11[−2.15, −0.07]	87.57	
**Ethnicity[c]**					0.11
East Asian	4	753	−0.43[−2.07, 1.22]	88.42	
Middle Eastern	1	104	−3.57[−9.80, −2.66]	NA	
South Asian	1	1601	0.00 [−1.74, 1.74]	NA	
White	2	133	−2.34[−3.66, −1.02]	38.81	
**Income level[d]**	4				0.11
Average or above:	5	677	−1.83[−2.94, −0.73]	72.91	
Below average	3	664	−0.07[−1.93, 1.80]	89.99	

[a] PROGRESS (Place of Residence, Race/ethnicity/culture/language, Occupation, Gender/sex, Religion, Education, Socioeconomic status, and Social)

[b] Thirteen studies reported educational status.

[c]Studies that did not report on ethnicity and those with mixed ethnicity were excluded. East Asian: three studies from China and one from South Korea. White: two from Ireland and two from Australia. South Asian: from India and Middle Eastern from Israel, including Jews and Bedouins.

[d] Mean income level for the country during the same year in which the study was conducted was taken as a cut-off point; Not applicable.

Note: subgroup analysis for gender, religion and social capital, could not be performed (Religion and social capital: not reported; Gender NA). There were no studies from low-income country, Africa, South America or the Pacific.

Table S5. Subgroup analyses of the effect of lifestyle intervention in women with a history of gestational diabetes on BMI by PROGRESS[a] framework

Analyses	Studies	Participants	Mean difference, kg (95% CI)	I^2	p-value for subgroup difference
**Country**					0.28
High-income	10	527	−0.60[−1.07, −0.13]	42.06	
Middle-income	5	1059	0.26[−1.23, 1.75]	98.90	
**Continent**					0.91
Asia	6	1163	0.02[−1.38, 1.42]	98.66	
Australia	4	129	−0.44[−1.13, 0.25]	34.14	
Europe	2	80	−0.67[−2.52, 1.18]	75.69	
North America	3	214	−0.55[−1.59, 0.48]	68.78	
**Educational status[b]**					0.31
Mostly without tertiary education	1	104	−1.61[−3.87, 0.65]	NA	
Mostly with tertiary education	8	1189	−0.40[−1.00, 0.21]	88.87	
**Ethnicity[c]**					0.19
East Asian	4	982	0.65[−0.95, −2.25]	99.15	
Middle Eastern	1	104	−1.61[−3.87, 0.65]	NA	
White	3	103	−0.79[−1.66, −0.07]	0.0	
**Income level[d]**					0.64
Average or above:	4	1041	−0.48[−1.16, 0.19]	58.07	
Below average	3	312	−0.14[−1.44, 1.16]	91.35	

[a] PROGRESS (Place of Residence, Race/ethnicity/culture/language, Occupation, Gender/sex, Religion, Education, Socioeconomic status, and Social)

[b] Eight studies reported educational status.

[c]Studies that did not report on ethnicity and those with mixed ethnicity were excluded. East Asian: All from China. White: two from Ireland and one from Australia. Middle Eastern from Israel, including Jews and Bedouins.

[d] Mean income level for the country during the same year in which the study was conducted was taken as a cut-off point; Not applicable.

Note: subgroup analysis for gender, religion and social capital, could not be performed (Religion and social capital: not reported; Gender NA). There was no study from low-income country, Africa, South America or the Pacific.

Figure S1. Funnel plots for publication bias of studies testing the effect of lifestyle intervention on Type 2 diabetes and body weight

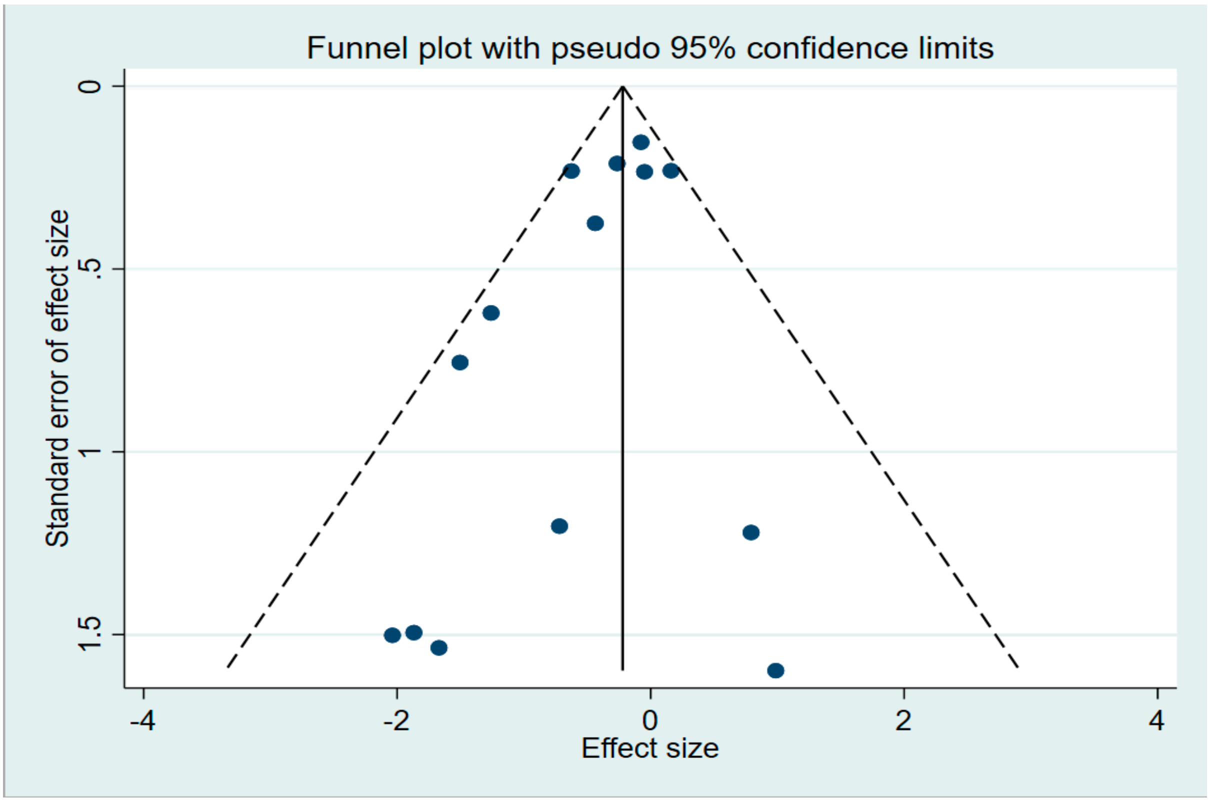

A) Type 2 diabetes: Egger's test P = 0.090

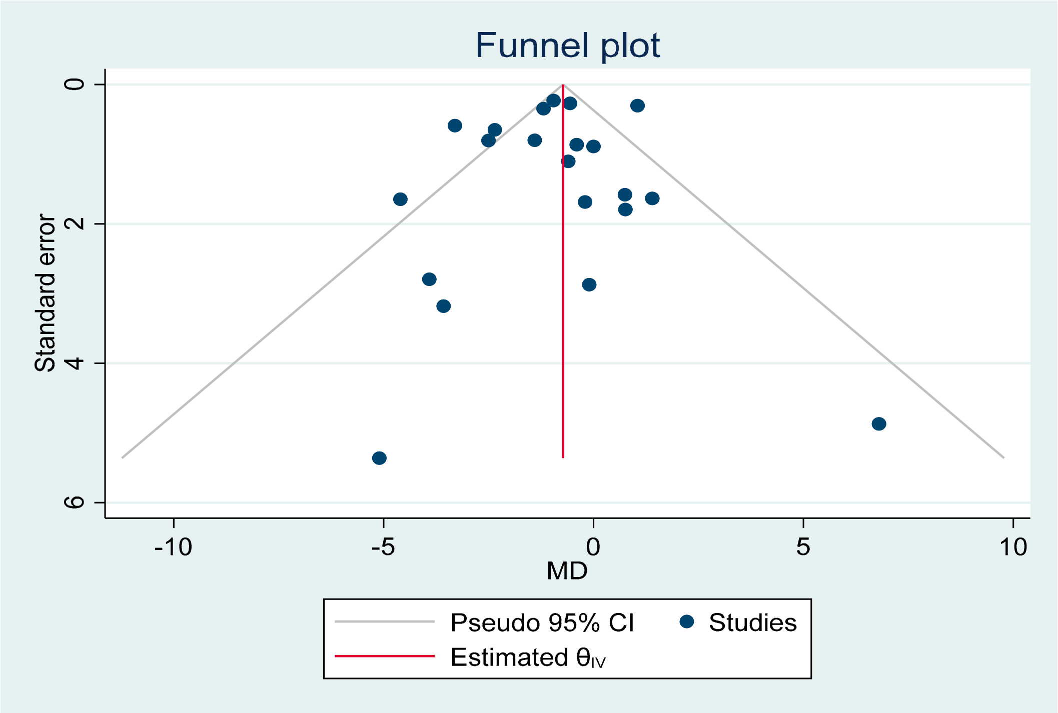

B) Body weight: Egger's test P = 0.995

Figure S2. Forest plots

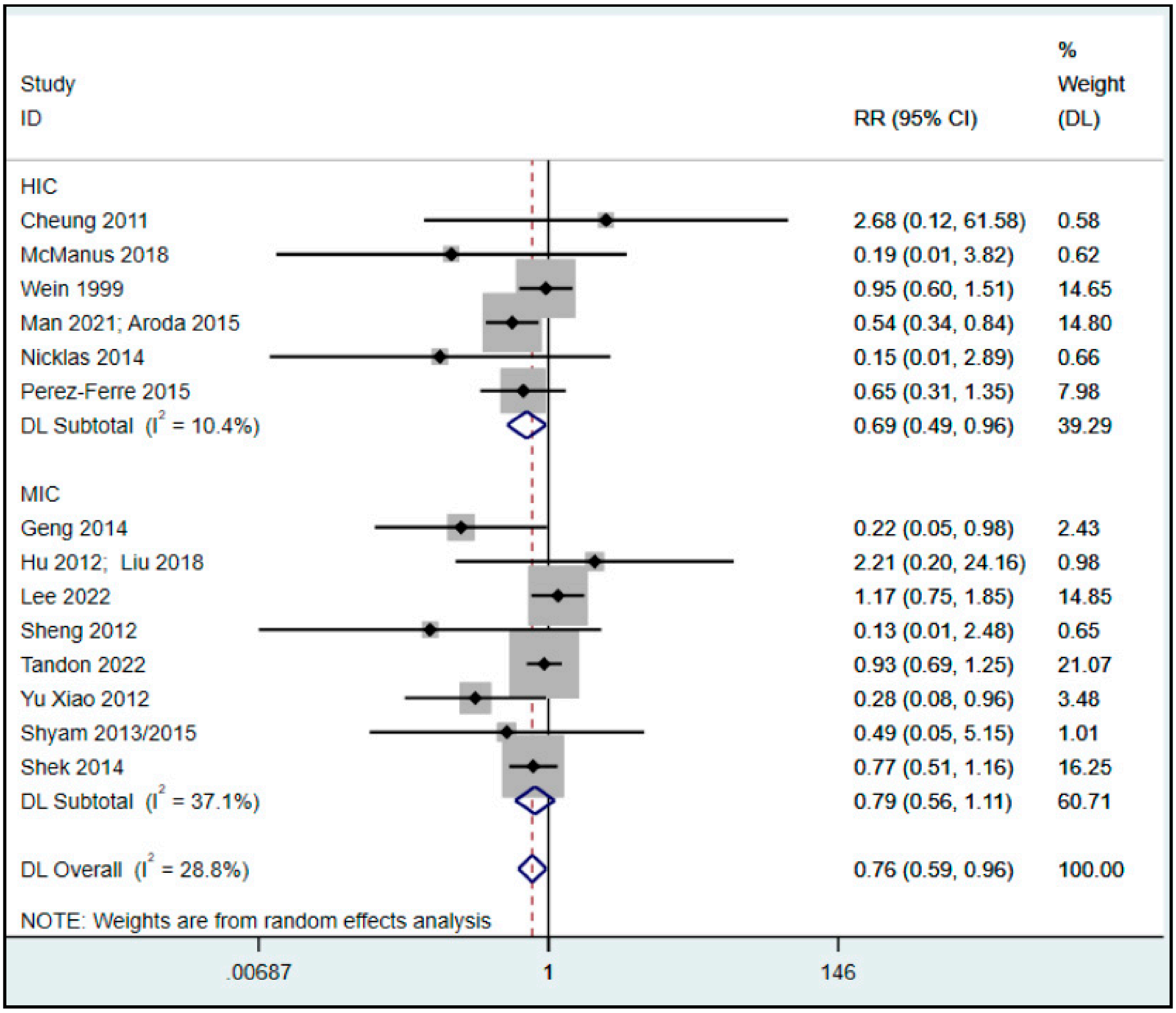

Figure S2.1. The effect of lifestyle intervention on T2DM by country according to the World Bank classification (subgroup differences: p-value=0.556)[35, 38, 47, 49, 51, 52, 58, 65, 72, 74-79].

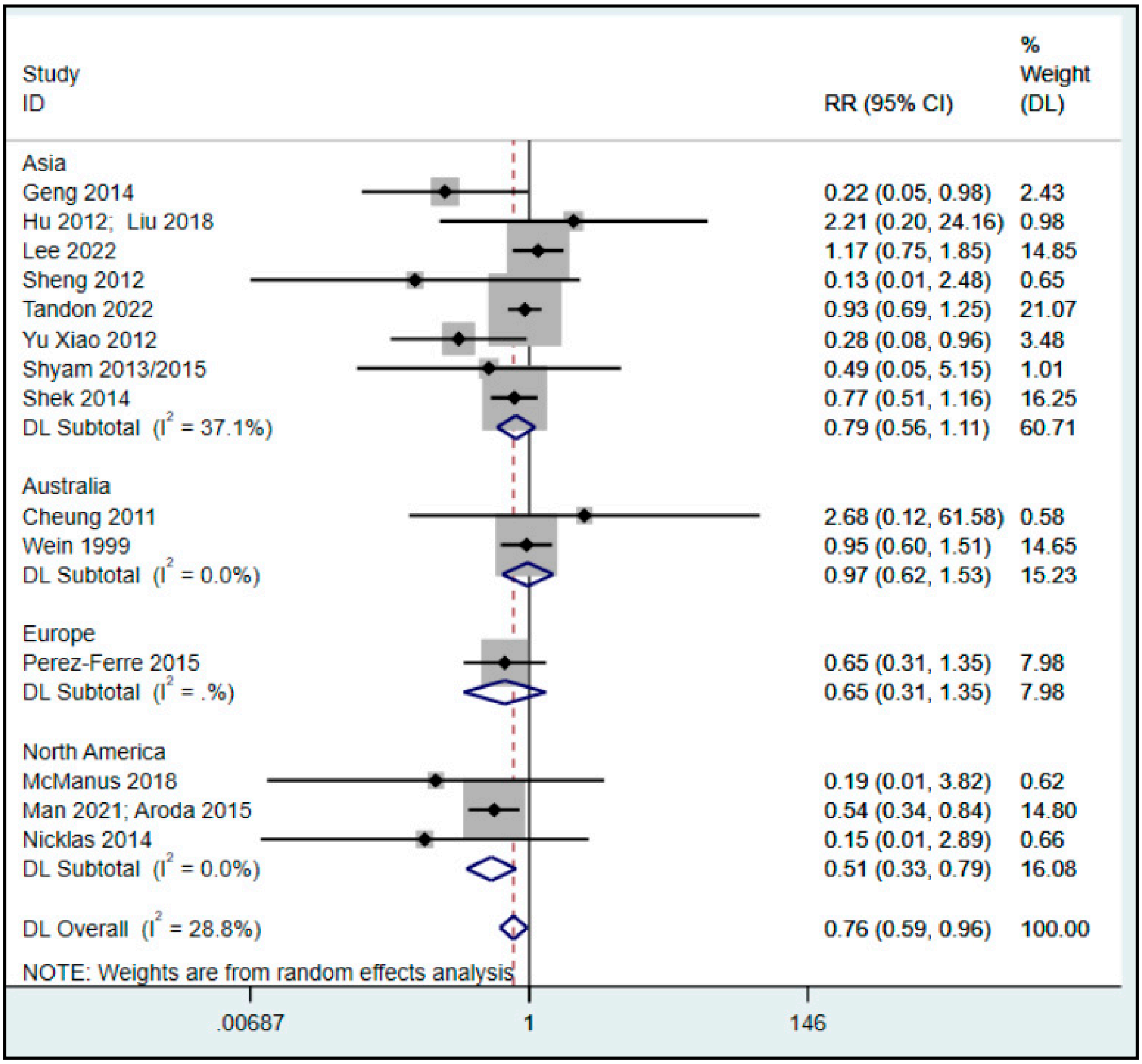

Figure S2.2. The effect of lifestyle intervention on T2DM by continent (subgroup differences: p-value=0.219) [35, 38, 47, 49, 51, 52, 58, 65, 72, 74-79].

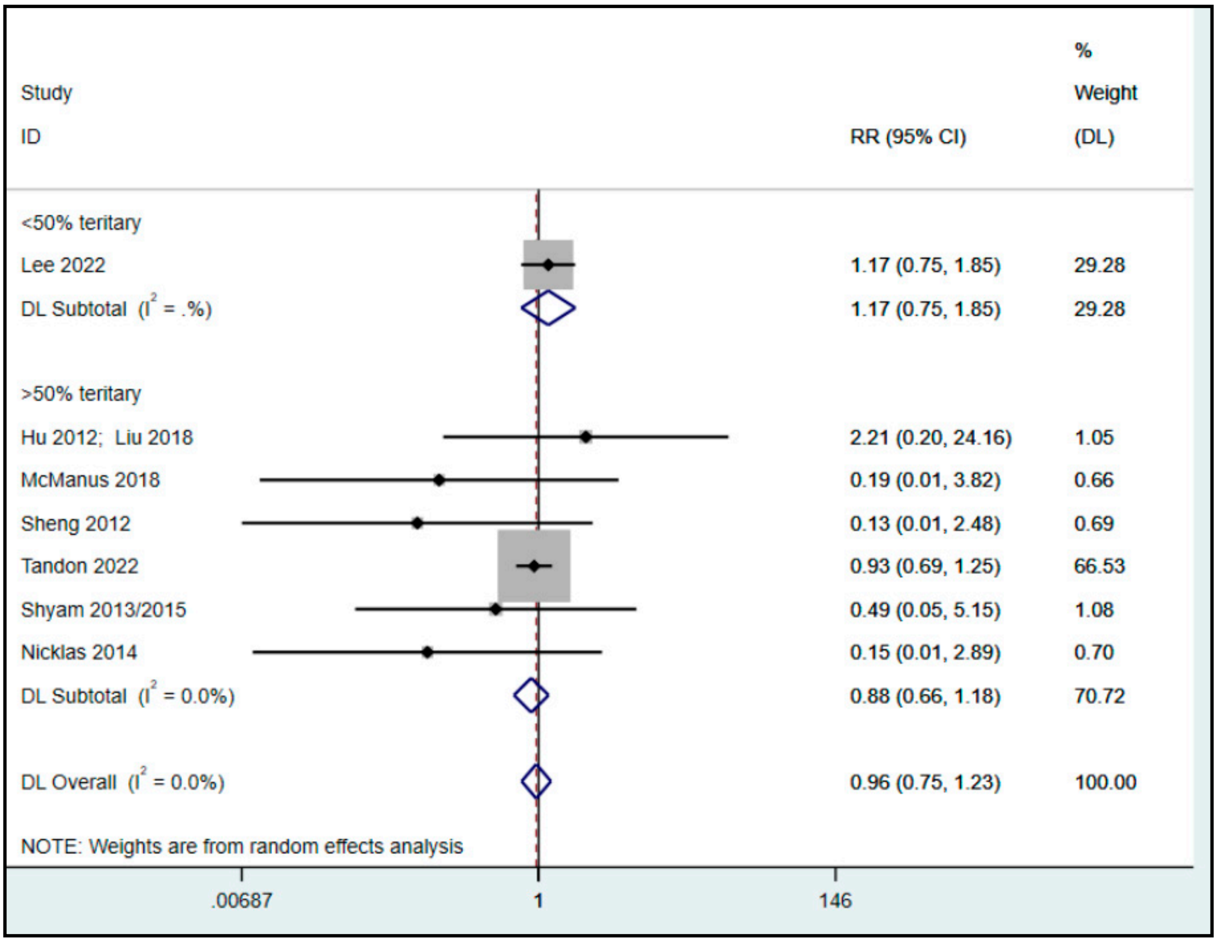

Figure S2.3. The effect of lifestyle intervention on T2DM by educational status. (subgroup differences: p-value=0.298) [35, 38, 51, 52, 61, 65, 75-77].

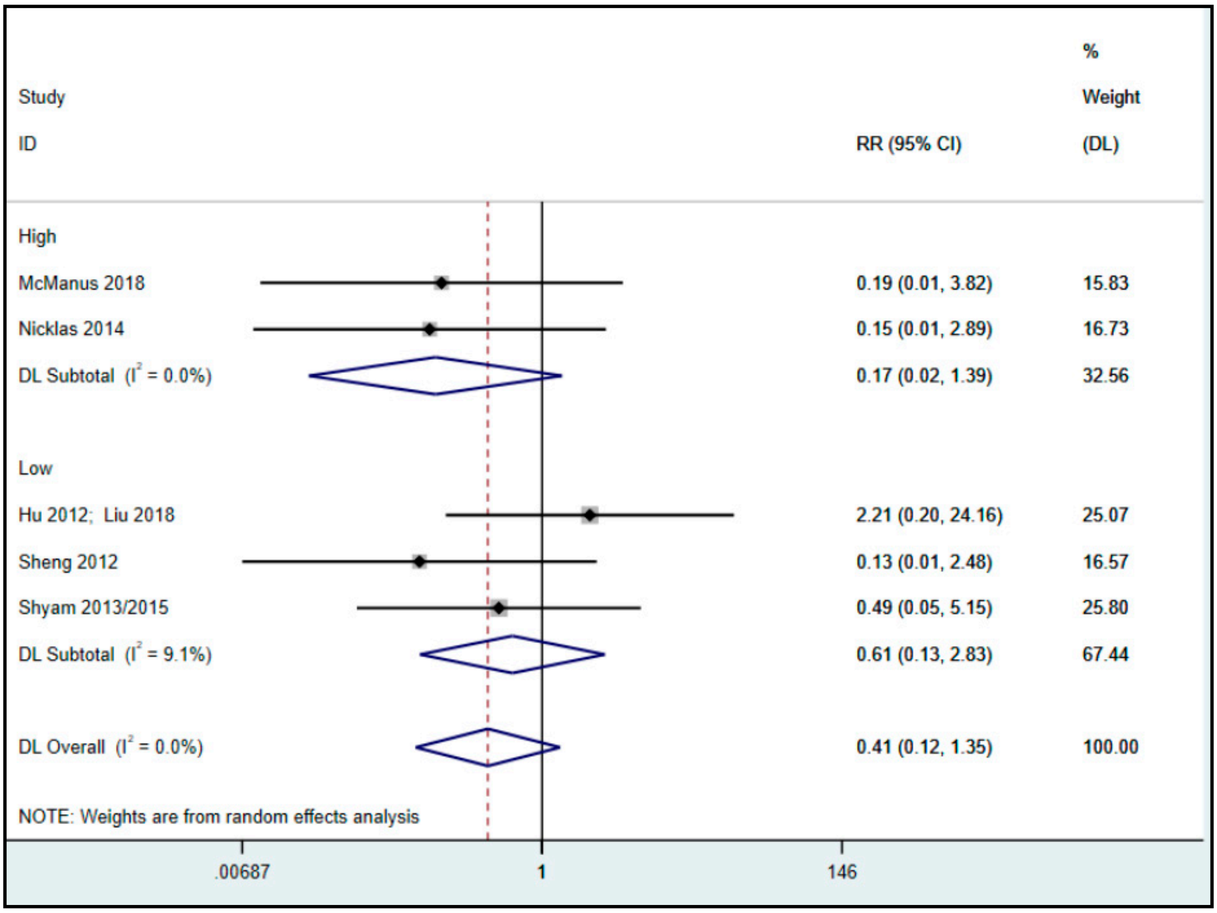

Figure S2.4. The effect of lifestyle intervention on T2DM by income. (subgroup differences: p-value=0.336) [35, 38, 51, 52, 61, 65, 75-77].

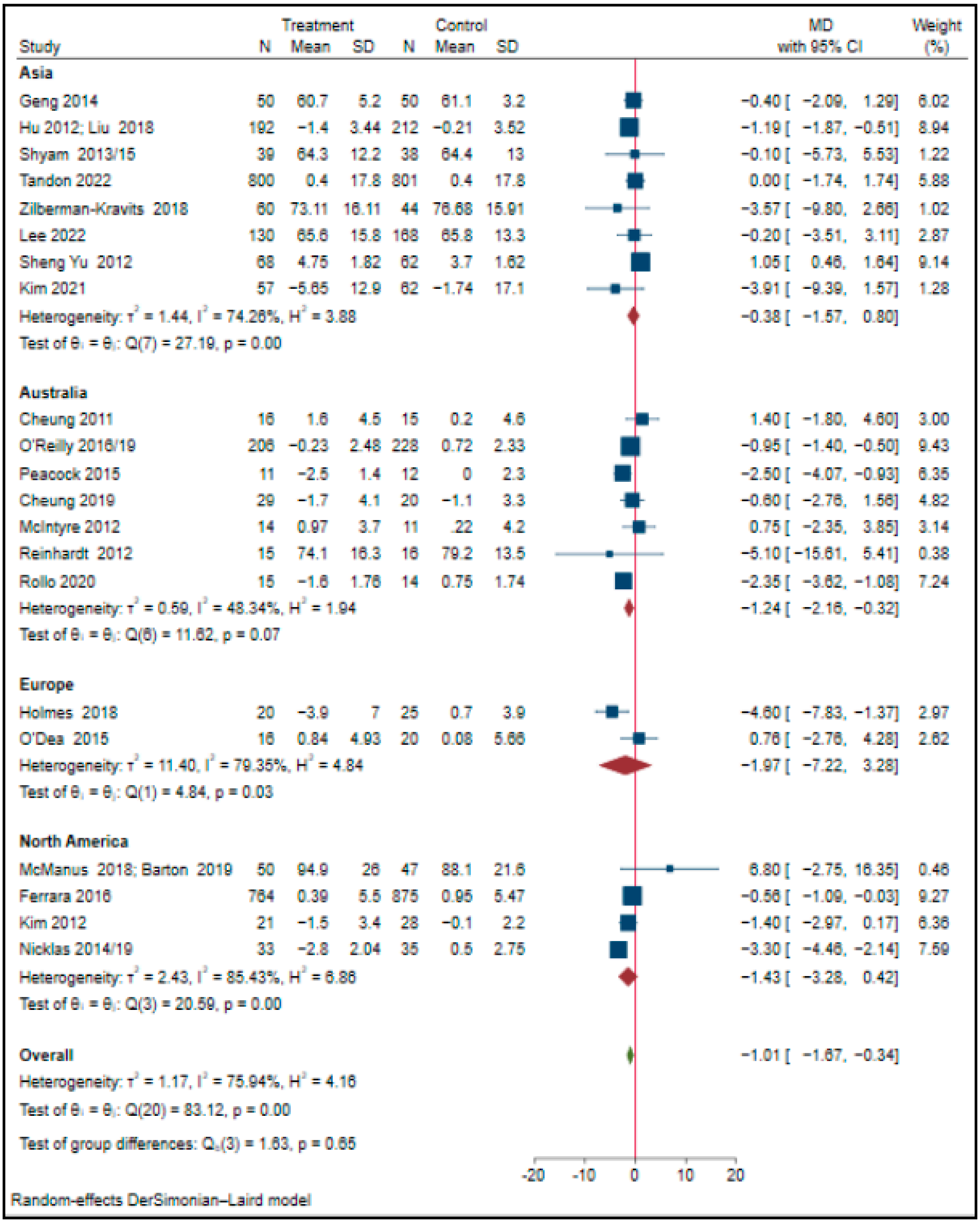

Figure S2.5. The effect of lifestyle intervention on body weight by continent [29, 32, 33, 35, 38, 44, 49-54, 58, 60, 61, 63-65, 68, 69, 71, 75-77, 80].

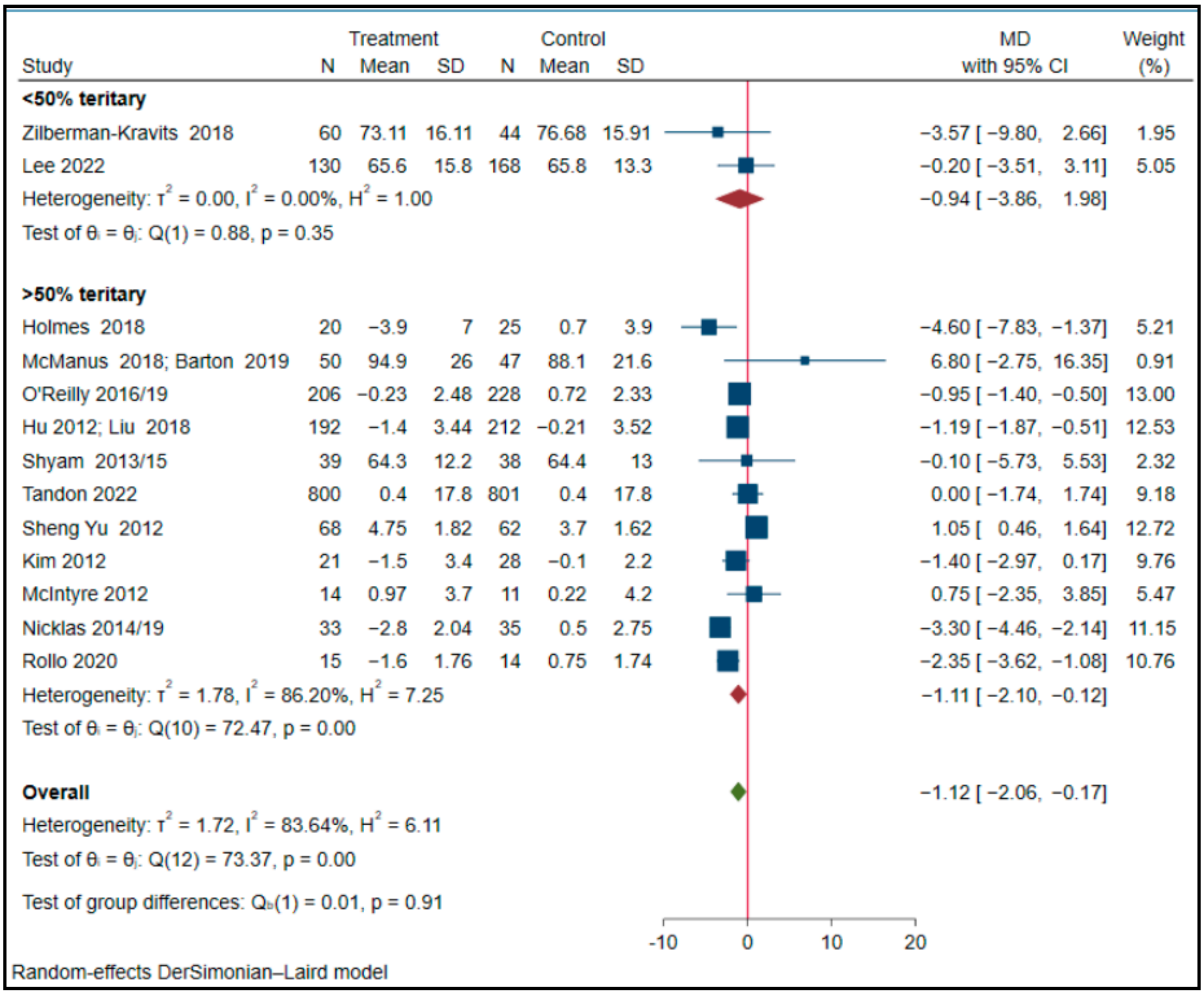

Figure S2.6. The effect of lifestyle intervention on body weight by educational status [29, 33, 35, 38, 50-52, 61, 63, 65, 68, 69, 71, 75-77, 80].

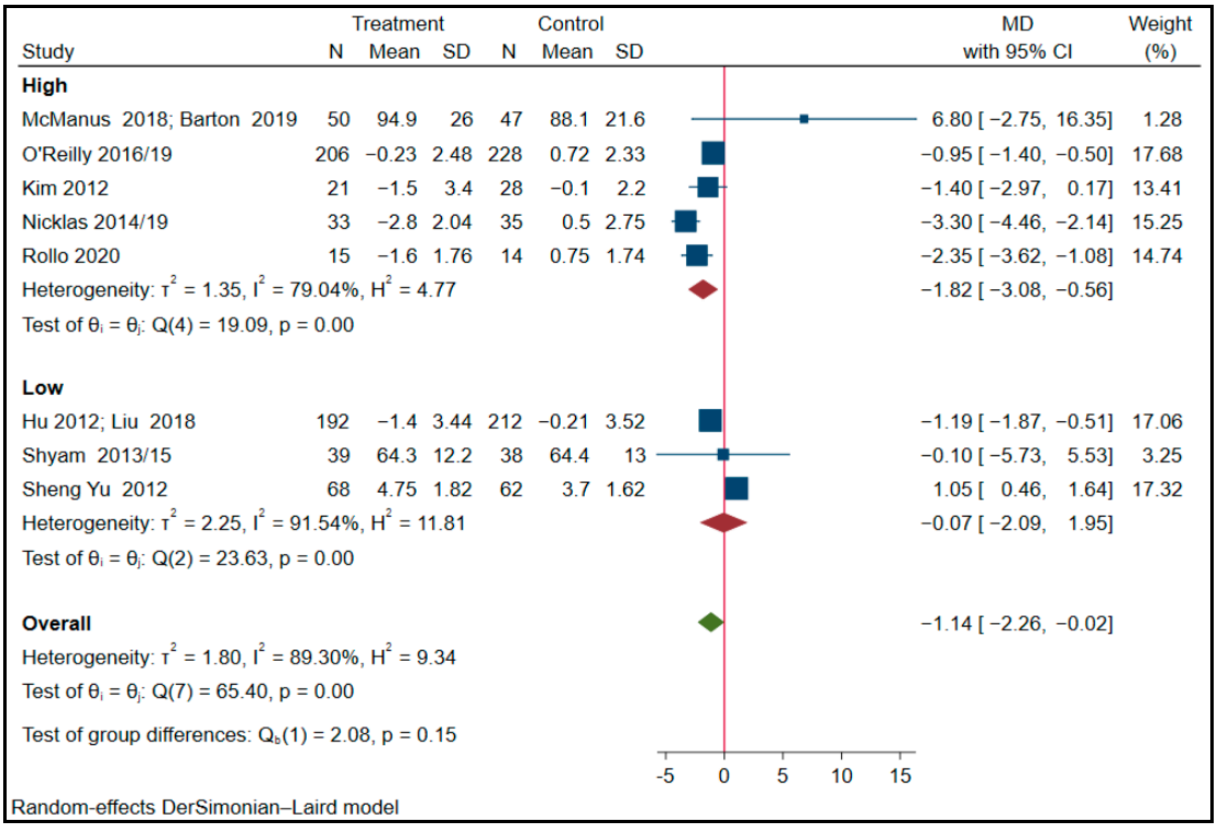

Figure S2.7. The effect of lifestyle intervention on body weight by income [29, 33, 35, 51, 52, 61, 63, 69, 71, 75-77].

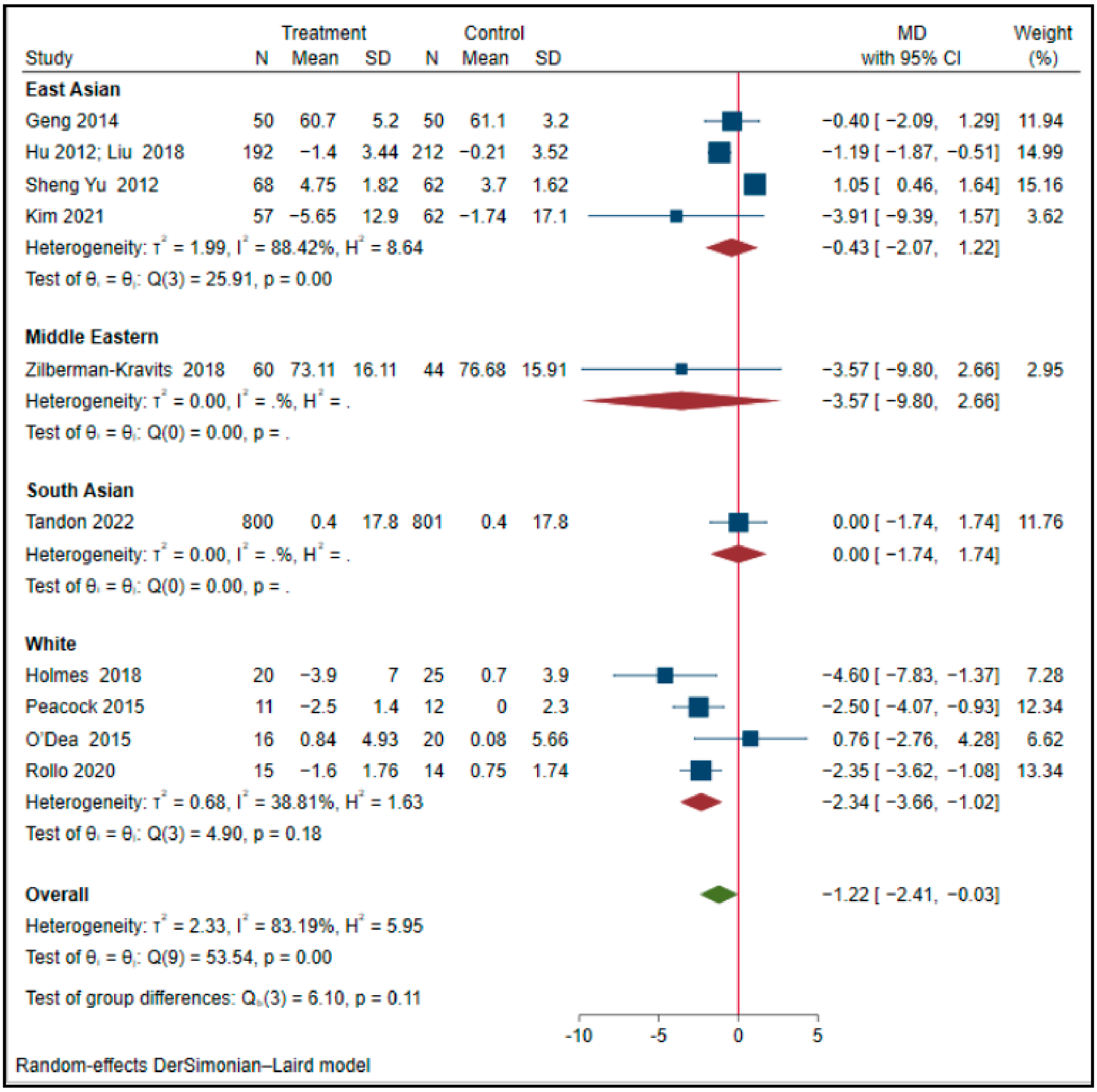

Figure S2.8. The effect of lifestyle intervention on body weight by ethnicity (Studies with mixed ethnicity and those with unknown ethnicity were excluded) [29, 35, 38, 49, 50, 53, 54, 61, 64, 75, 80].

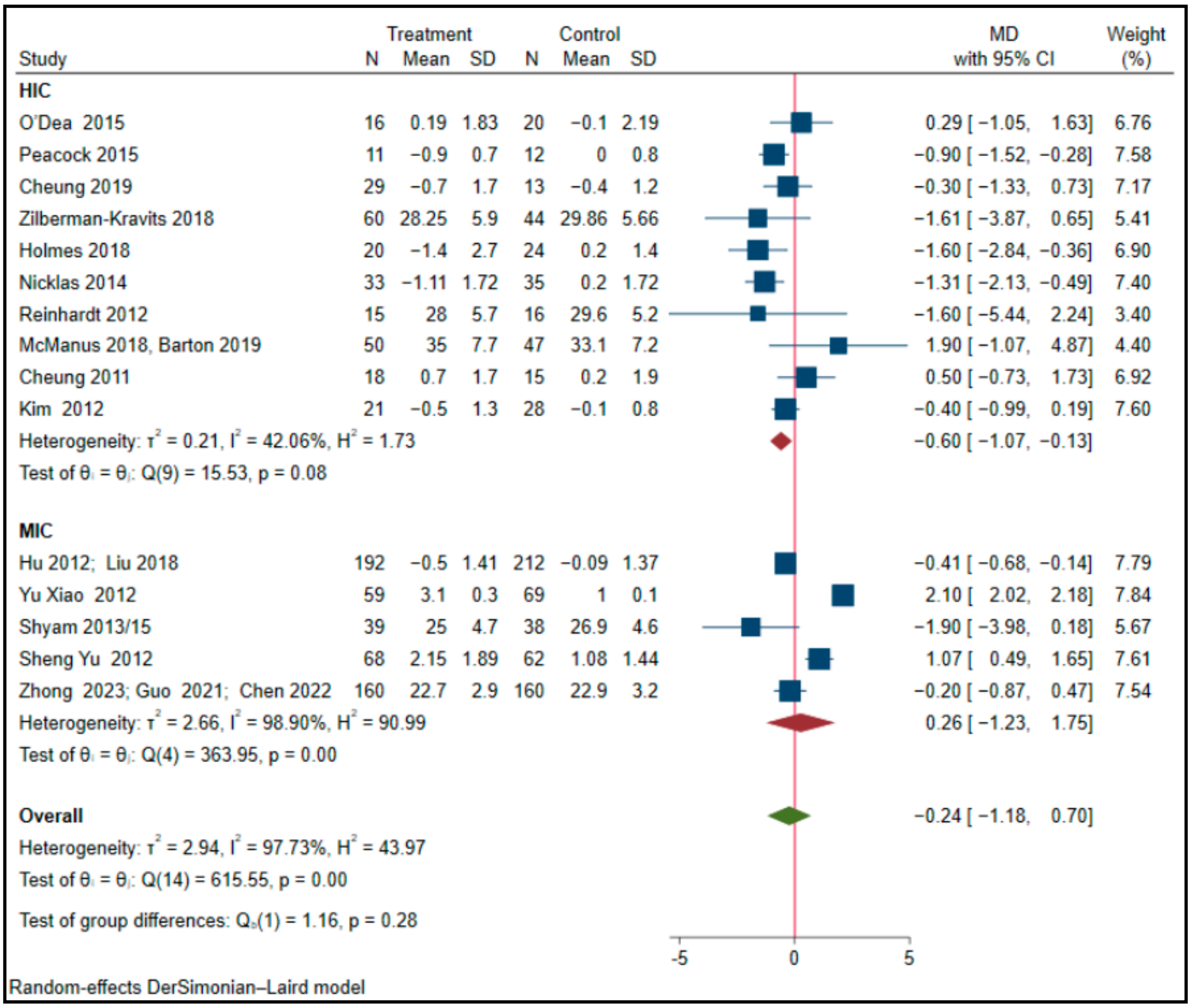

Figure S2.9. The effect of lifestyle intervention on BMI by country income classification according to the World Bank [32, 35, 43-46, 50-54, 58, 61, 63, 69, 75-77, 79, 80].

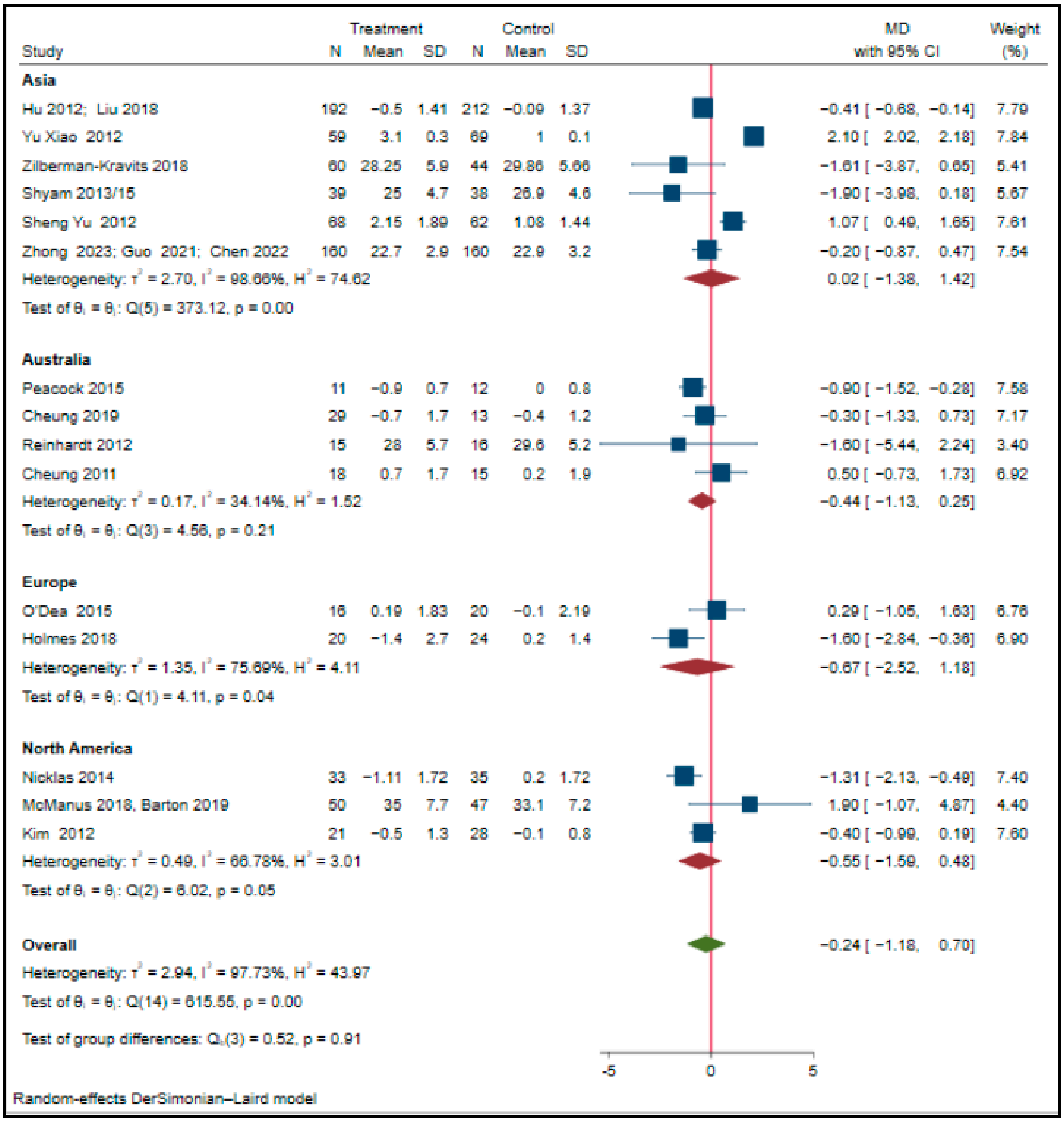

Figure S2.10. The effect of lifestyle intervention on BMI by continent [29, 32, 33, 35, 38, 44, 49-54, 58, 60, 61, 63-65, 68, 71, 75-77, 80].

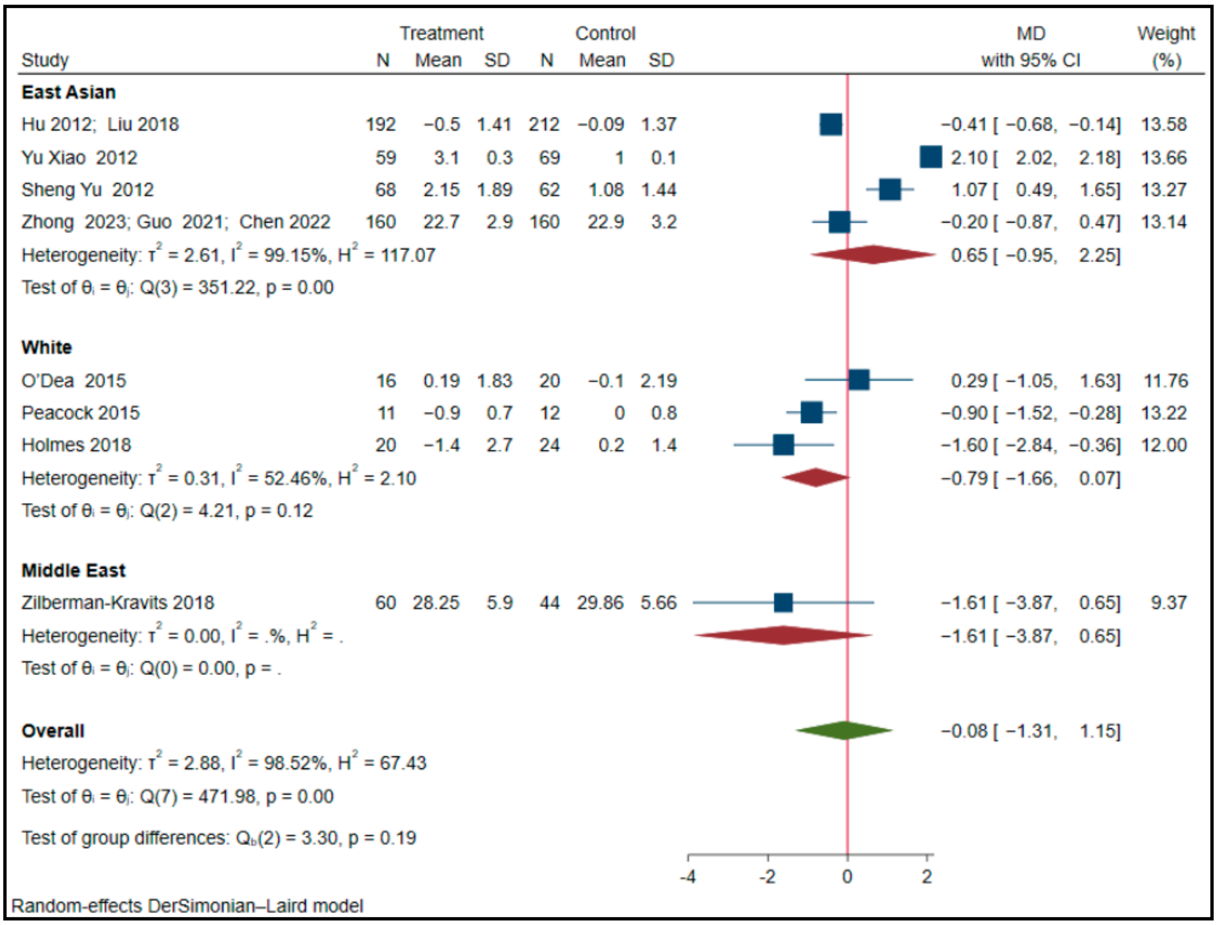

Figure S2.11. The effect of lifestyle intervention on BMI by ethnicity Studies with mixed ethnicity and those with unknown ethnicity were excluded) [35, 43, 45, 46, 50, 53, 54, 61, 75, 79, 80].

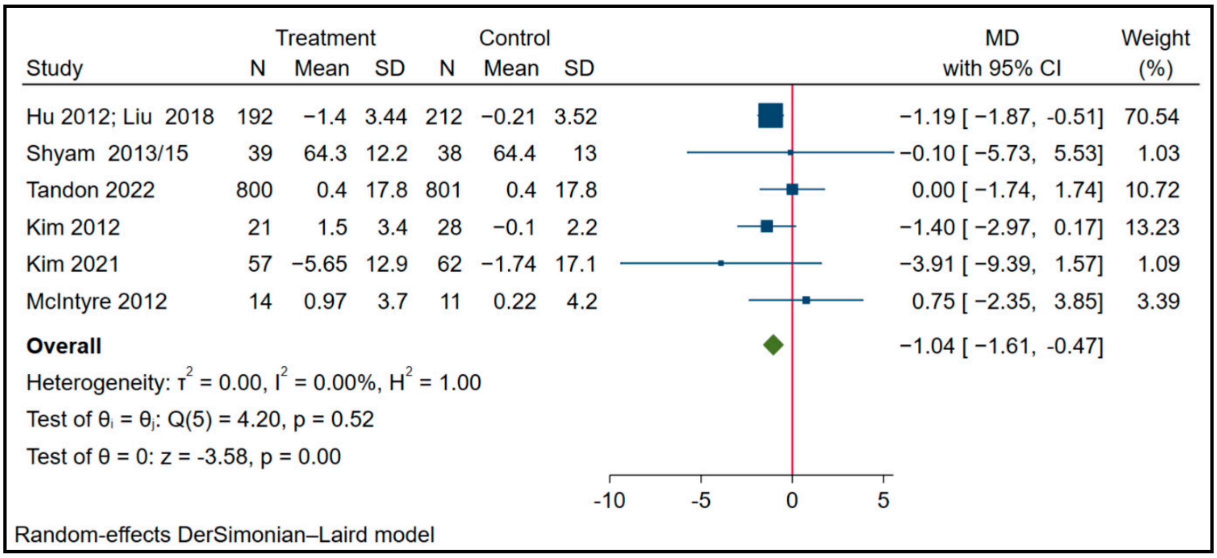

Figure S2.12 Sensitivity analysis (The effect of lifestyle intervention on body weight after excluding studies with a high risk of bias [35, 38, 61, 63, 64, 68, 76, 77].

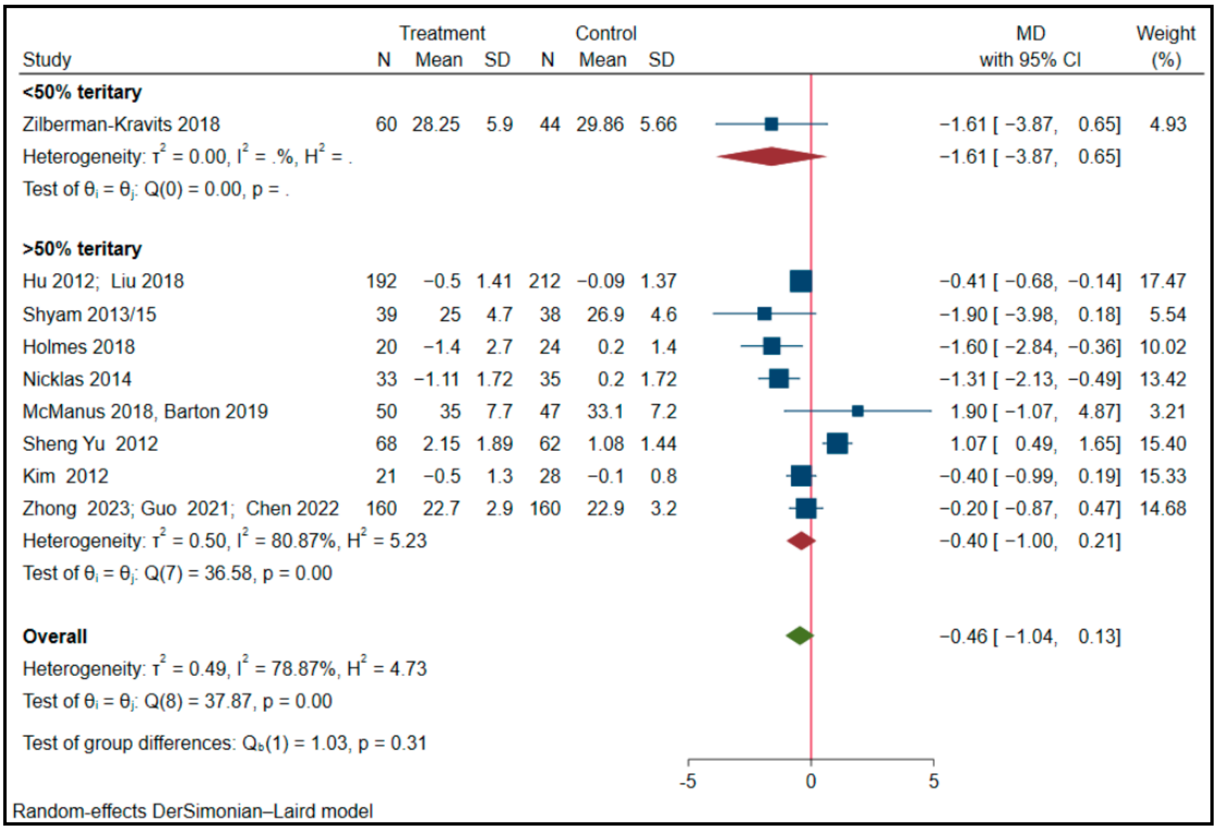

Figure S2.13. The effect of lifestyle intervention on BMI by educational status [35, 43, 45, 46, 50-52, 61, 63, 69, 75-77, 80]

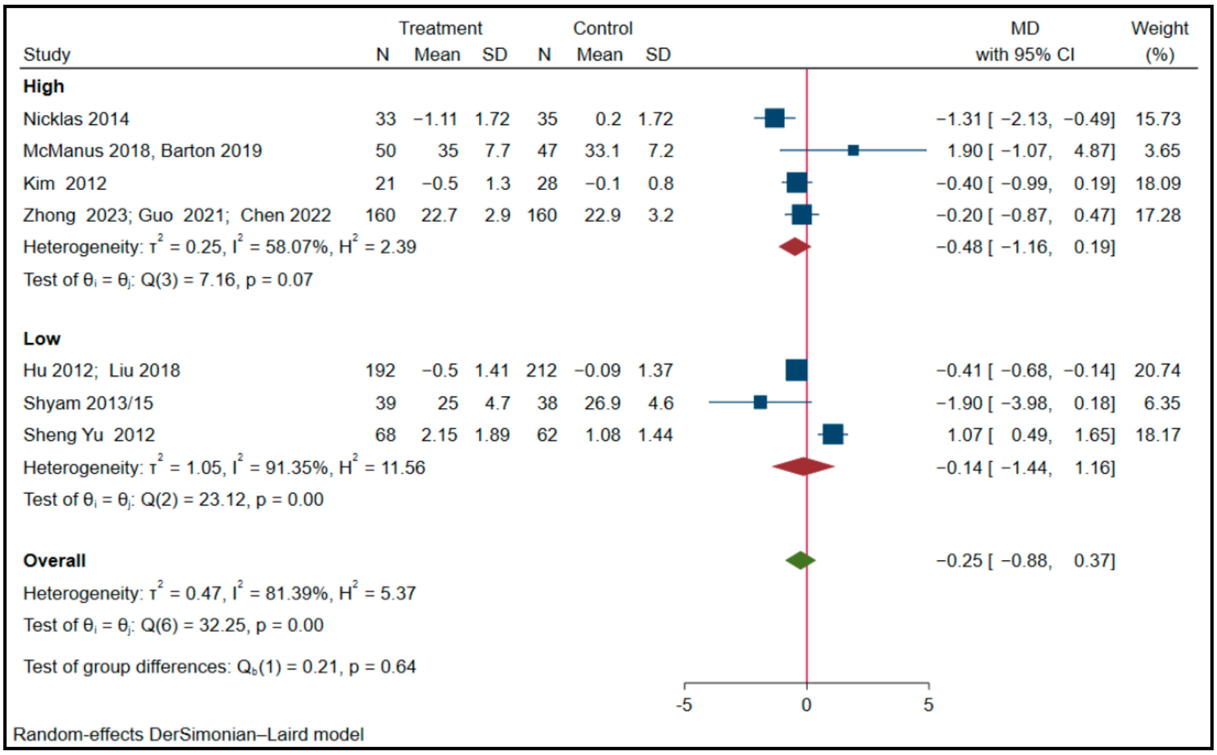

Figure S2.14. The effect of lifestyle intervention on BMI by income [35, 43, 45, 46, 51, 52, 61, 63, 69, 75-77]

References:

1. Hill-Briggs, F.; Adler, N.E.; Berkowitz, S.A.; Chin, M.H.; Gary-Webb, T.L.; Navas-Acien, A.; Thornton, P.L.; Haire-Joshu,,D. Social determinants of health and diabetes: A scientific review. *Diabetes Care* **2021**, *44*, 258–279.
2. IDF. *Diabetes Atlas*, 10th ed.; IDF: Springfield, MO, USA, 2021.
3. Sommer, I.; Griebler, U.; Mahlknecht, P.; Thaler, K.; Bouskill, K.; Gartlehner, G.; Mendis S. Socioeconomic inequalities in non-communicable diseases and their risk factors: An overview of systematic reviews. *BMC Public Health* **2015**, *15*, 914.
4. Agardh, E.; Allebeck, P.; Hallqvist, J.; Moradi, T.; Sidorchuk, A. Type 2 diabetes incidence and socio-economic position: A systematic review and meta-analysis. *Int. J. Epidemiol.* **2011**, *40*, 804–818.
5. Peykari, N.; Djalalinia, S.; Qorbani, M.; Sobhani, S.; Farzadfar, F.; Larijani, B. Socioeconomic inequalities and diabetes: A systematic review from Iran. *J. Diabetes Metab. Disord.* **2015**, *14*, 8.
6. Maty, S.C.; Everson-Rose, S.A.; Haan, M.N.; Raghunathan, T.E.; Kaplan, G.A. Education, income, occupation, and the 34-year incidence (1965–99) of type 2 diabetes in the Alameda County Study. *Int. J. Epidemiol.* **2005**, *34*, 1274–1281.
7. Tung, E.L.; Baig, A.A.; Huang, E.S.; Laiteerapong, N.; Chua, K.-P. Racial and ethnic disparities in diabetes screening between Asian Americans and other adults: BRFSS 2012–2014. *J. Gen. Intern. Med.* **2017**, *32*, 423–429.
8. Wang, H.; Li, N.; Chivese, T.; Werfalli, M.; Sun, H.; Yuen, L.; Hoegfeldt, C.A.; Powe, C.C.; Immanuel, J.; Karuranga, S.; et al. IDF diabetes atlas: Estimation of global and regional gestational diabetes mellitus prevalence for 2021 by International Association of Diabetes in Pregnancy Study Group's Criteria. *Diabetes Res. Clin. Pract.* **2022**, *183*, 109050.
9. Yuen, L.; Wong, V.W. Gestational diabetes mellitus: Challenges for different ethnic groups. *World J. Diabetes* **2015**, *6*, 1024.
10. Shah, N.S.; Wang, M.C.; Freaney, P.M.; Perak, A.M.; Carnethon, M.R.; Kandula, N.R.; Gunderson, E.P.; Bullard, K.M; Grobman, W.A.; O'Brien, M.J.; et al. Trends in gestational diabetes at first live birth by race and ethnicity in the US, 2011–2019. *JAMA* **2021**, *326*, 660–669.
11. Vounzoulaki, E.; Khunti, K.; Abner, S.C.; Tan, B.K.; Davies, M.J.; Gillies, C.L. Progression to type 2 diabetes in women with a known history of gestational diabetes: Systematic review and meta-analysis. *BMJ* **2020**, *369*, m1361.
12. CDC. NCHHSTP. social determinants of health. *Retrieved March* **2014**, *8*, 2016.
13. Currie, C.; Zanotti, C.; Morgan, A.; Currie, D.; De Looze, M.; Roberts, C.; Samdal, O.; Smith, O.R.F; Barnekow, V. Social determinants of health and well-being among young people. *Health Behav. Sch.-Aged Child. (HBSC) Study Int. Rep.* **2009**, *2010*, 271.
14. Braveman, P.; Gottlieb, L. The social determinants of health: it's time to consider the causes of the causes. *Public Health Rep.* **2014**, *129* (Suppl. 2), 19–31.
15. Girgis, C.M.; Gunton, J.E.; Cheung, N.W. The influence of ethnicity on the development of type 2 diabetes mellitus in women with gestational diabetes: A prospective study and review of the literature. *Int. Sch. Res. Not.* **2012**, *2012*, 341638.
16. Marmot, M.; Friel, S.; Bell, R.; Houweling, T.A.; Taylor, S.; on behalf of the Commission on Social Determinants of Health. Closing the gap in a generation: Health equity through action on the social determinants of health. *Lancet* **2008**, *372*, 1661–1669.
17. Lorenc, T.; Petticrew, M.; Welch, V.; Tugwell, P. What types of interventions generate inequalities? Evidence from systematic reviews. *J. Epidemiol. Community Health* **2013**, *67*, 190–193.
18. Chapman, A.R.; Carbonetti, B. Human rights protections for vulnerable and disadvantaged groups: The contributions of the UN Committee on Economic, Social and Cultural Rights. *Hum. Rights Q.* **2011**, *33*, 682.
19. Hart, J.T. The inverse care law. *Lancet* **1971**, *297*, 405–412.
20. Gordon, D.; Shaw, M. *Inequalities in Health: The Evidence Presented to the Independent Inquiry into Inequalities in Health, Chaired by Sir Donald Acheson*; Policy Press: Bristol, UK, 1999.
21. Chatzi, G.; Whittaker, W.; Chandola, T.; Mason, T.; Soiland-Reyes, C.; Sutton, M.; Bower, P. Diabetes Prevention Programme and socioeconomic inequalities in Type 2 Diabetes in England: Georgia Chatzi. *Eur. J. Public Health* **2022**, *32* (Suppl. 3), ckac129–ckac159.
22. Welch, V.A.; Petkovic, J.; Jull, J.; Hartling, L.; Klassen, T.; Kristjansson, E.; Pardo, J.P.; Petticrew, M.; Stott, D.J.; Thomson, D.; et al. Equity and specific populations. In *Cochrane Handbook for Systematic Reviews of Interventions;* John Wiley and Sons: Hoboken, NJ, USA, 2019; pp. 433–449.
23. O'Neill, J.; Tabish, H.; Welch, V.; Petticrew, M.; Pottie, K.; Clarke, M.; Evans, T.; Pardo, J.P.; Waters, E.; White, H.; et al. Applying an equity lens to interventions: Using PROGRESS ensures consideration of socially stratifying factors to illuminate inequities in health. *J. Clin. Epidemiol.* **2014**, *67*, 56–64.

24. Page, M.J.; McKenzie, J.E.; Bossuyt, P.M.; Boutron, I.; Hoffmann, T.C.; Mulrow, C.D.; Shamseer , L.; Tetzlaff, J.M.; Akl, E.A.; Brennan, S.E.; et al. The PRISMA 2020 statement: An updated guideline for reporting systematic reviews. *Syst. Rev.* **2021**, *10*, 89.
25. Sterne, J.A.; Savović, J.; Page, M.J.; Elbers, R.G.; Blencowe, N.S.; Boutron, I.; Cates, C.J.; Cheng, H.Y.; Corbett, M.S.; Eldridge, S.M.; et al. RoB 2: A revised tool for assessing risk of bias in randomised trials. *BMJ* **2019**, *366*, l4898.
26. Sterne, J.A.; Hernán, M.A.; Reeves, B.C.; Savović, J.; Berkman, N.D.; Viswanathan, M.; Henry, D.; Altman, D.G.; Ansari, M.T.; Boutron, I.; et al. ROBINS-I: A tool for assessing risk of bias in non-randomised studies of interventions. *BMJ* **2016**, *355*, i4919.
27. Peterson, J.; Welch, V.; Losos, M.; Tugwell, P. The Newcastle-Ottawa scale (NOS) for assessing the quality of nonrandomised studies in meta-analyses. *Ott. Ott. Hosp. Res. Inst.* **2011**, *2*, 1–12.
28. Fantom, N.J.; Serajuddin, U. *The World Bank's Classification of Countries by Income*; World Bank Policy Research Working Paper; World Bank Group: Washington, DC, USA, 2016; p. 7528.
29. Rollo, M.E.; Baldwin, J.N.; Hutchesson, M.; Aguiar, E.J.; Wynne, K.; Young, A.; Callister, R.; Haslam, R.; Clare E Collins, C.E. The feasibility and preliminary efficacy of an eHealth lifestyle program in women with recent gestational diabetes mellitus: A pilot study. *Int. J. Environ. Res. Public Health* **2020**, *17*, 7115.
30. McCurley, J.L.; Fortmann, A.L.; Gutierrez, A.P.; Gonzalez, P.; Euyoque, J.; Clark, T.; Preciado, J.; Ahmad, A.; Philis-Tsimikas, A.; Gallo, L.C. Pilot test of a culturally appropriate diabetes prevention intervention for at-risk Latina women. *Diabetes Educ.* **2017**, *43*, 631–640.
31. Philis-Tsimikas, A.; Fortmann, A.L.; Dharkar-Surber, S.; Euyoque, J.A.; Ruiz, M.; Schultz, J.; Gallo, L.C. Dulce Mothers: An intervention to reduce diabetes and cardiovascular risk in Latinas after gestational diabetes. *Transl. Behav. Med.* **2014**, *4*, 18–25.
32. Cheung, N.W.; Blumenthal, C.; Smith, B.J.; Hogan, R.; Thiagalingam, A.; Redfern, J.; Barry, T.; Cinnadaio, N.; Chow, C.K. A pilot randomised controlled trial of a text messaging intervention with customisation using linked data from wireless wearable activity monitors to improve risk factors following gestational diabetes. *Nutrients* **2019**, *11*, 590.
33. O'Reilly, S.L.; Dunbar, J.A.; Versace, V.; Janus, E.; Best, J.D.; Carter, R.; Oats, J.J.N.; Skinner, T.; Ackland, M.; Phillips, P.A.; et al. Mothers after Gestational Diabetes in Australia (MAGDA): A randomised controlled trial of a postnatal diabetes prevention program. *PLoS Med.* **2016**, *13*, e1002092.
34. Chen, M.; Ukke, G.G.; Moran, L.J.; Sood, S.; Bennett, C.J.; Bahri Khomami, M.; Absetz, P.; Teede, H.; Harrison, C.L.; Lim, S. The effect of lifestyle intervention on diabetes prevention by ethnicity: A systematic review of intervention characteristics using the tidier framework. *Nutrients* **2021**, *13*, 4118.
35. Hu, G.; Tian, H.; Zhang, F.; Liu, H.; Zhang, C.; Zhang, S.; Wang, L.; Liu, G.; Yu, Z.; Yang, X.; et al. Tianjin Gestational Diabetes Mellitus Prevention Program: Study design, methods, and 1-year interim report on the feasibility of lifestyle intervention program. *Diabetes Res. Clin. Pract.* **2012**, *98*, 508–517.
36. Higgins, J.P.; Thompson, S.G.; Deeks, J.J.; Altman, D.G. Measuring inconsistency in meta-analyses. *BMJ* **2003**, *327*, 557–560.
37. StataCorp LLC. *Stata Statistical Software: Release Vol. 16*; StataCorp LLC: College Station, TX, USA, 2019.
38. Tandon, N.; Gupta, Y.; Kapoor, D.; Lakshmi, J.K.; Praveen, D.; Bhattacharya, A.; et al. Effects of a Lifestyle Intervention to Prevent Deterioration in Glycemic Status Among South Asian Women With Recent Gestational Diabetes: A Randomized Clinical Trial. *JAMA Netw. Open* 2022, 5, e220773.
39. Lim, S.; Dunbar, J.A.; Versace, V.L.; Janus, E.; Wildey, C.; Skinner, T.; O'Reilly, S. Comparing a telephone-and a group-delivered diabetes prevention program: Characteristics of engaged and non-engaged postpartum mothers with a history of gestational diabetes. *Diabetes Res. Clin. Pract.* **2017**, *126*, 254–262.
40. Li, M.; Lin, Q.; Shi, J.; Xi, Y.; Xiang, C.; Yong, C.; Guo, J. The Impact of Lifestyle Intervention on Dietary Quality among Rural Women with Previous Gestational Diabetes Mellitus—A Randomized Controlled Study. *Nutrients* **2021**, *13*, 2642.
41. Lim, K.; Chan, S.-Y.; Lim, S.L.; Tai, B.C.; Tsai, C.; Wong, S.R.; Ang, S.M.; Yew, T.W.; Tai, E.S.; Yong, E.L. A Smartphone App to Restore Optimal Weight (SPAROW) in Women With Recent Gestational Diabetes Mellitus: Randomized Controlled Trial. *JMIR Mhealth Uhealth* **2021**, *9*, e22147.
42. Brokaw, S.M.; Carpenedo, D.; Campbell, P.; Butcher, M.K.; Helgerson, S.D.; Harwell, T.S. Does a History of Gestational Diabetes Mellitus Affect Key Outcomes in Women Participating in a Diabetes Prevention Program? *Matern. Child Health J.* **2018**, *22*, 529–537.
43. Guo, J.; Long, Q.; Yang, J.; Lin, Q.; Wiley, J.; Chen, J.-L. The Efficacy of an Intensive Lifestyle Modification Program on Psychosocial Outcomes among Rural Women with Prior Gestational Diabetes Mellitus: Six Months Follow-Up of a Randomized Controlled Trial. *Int. J. Environ. Res. Public Health* **2021**, *18*, 1519.

44. Reinhardt, J.A.; Van Der Ploeg, H.P.; Grzegrzulka, R.; Timperley, J.G. Implementing lifestyle change through phone-based motivational interviewing in rural-based women with previous gestational diabetes mellitus. *Health Promot. J. Aust.* **2012**, *23*, 5–9.
45. Chen, Y.; Zhong, Q.; Luo, J.; Tang, Y.; Li, M.; Lin, Q.; Willey, J.A.; Chen, J.L; Whittemore, R.; Guo, J. The 6-month efficacy of an intensive lifestyle modification program on type 2 diabetes risk among rural women with prior gestational diabetes mellitus: A cluster randomized controlled trial. *Prev. Sci.* **2022**, *23*, 1156–1168.
46. Zhong, Q.; Chen, Y.; Luo, M.; Lin, Q.; Tan, J.; Xiao, S.; Willey, J.A.; Chen, J.L; Whittemore, R.; Guo, J. The 18-month efficacy of an Intensive LifeStyle Modification Program (ILSM) to reduce type 2 diabetes risk among rural women: A cluster randomized controlled trial. *Glob. Health* **2023**, *19*, 6.
47. Man, B.; Schwartz, A.; Pugach, O.; Xia, Y.; Gerber, B. A clinical diabetes risk prediction model for prediabetic women with prior gestational diabetes. *PLoS ONE* **2021**, *16*, e0252501.
48. Ferrara, A.; Hedderson, M.M.; Albright, C.L.; Ehrlich, S.F.; Quesenberry, C.P., Jr.; Peng, T.; Feng, J.; Ching, J.; Crites, Y. A pregnancy and postpartum lifestyle intervention in women with gestational diabetes mellitus reduces diabetes risk factors: A feasibility randomized control trial. *Diabetes Care* **2011**, *34*, 1519–1525.
49. Ling, G.; Shufeng, W.; Xiaoming, Z.; Suhua, Y. Discussion on postpartum lifestyle intervention plan for women with gestational diabetes mellitus. *Henan J. Surg.* **2014**, *20*, 76–78.
50. Holmes, V.A.; Draffin, C.R.; Patterson, C.C.; Francis, L.; Irwin, J.; McConnell, M.; Farrell, B.; Brennan, S.F.; McSorley, O.; Wotherspoon, A.C.; et al. Postnatal lifestyle intervention for overweight women with previous gestational diabetes: A randomized controlled trial. *J. Clin. Endocrinol. Metab.* **2018**, *103*, 2478–2487.
51. McManus, R.; Miller, D.; Mottola, M.; Giroux, I.; Donovan, L. Translating healthy living messages to postpartum women and their partners after gestational diabetes (GDM): Body habitus, A1c, lifestyle habits, and program engagement results from the families Defeating diabetes (FDD) randomized trial. *Am. J. Health Promot.* **2018**, *32*, 1438–1446.
52. Nicklas, J.M.; Zera, C.A.; England, L.J.; Rosner, B.A.; Horton, E.; Levkoff, S.E.; Seely, E.W. A web-based lifestyle intervention for women with recent gestational diabetes mellitus: A randomized controlled trial. *Obstet. Gynecol.* **2014**, *124*, 563.
53. Peacock, A.; Bogossian, F.E.; Wilkinson, S.; Gibbons, K.; Kim, C.; McIntyre, H. A randomised controlled trial to delay or prevent type 2 diabetes after gestational diabetes: Walking for exercise and nutrition to prevent diabetes for you. *Int. J. Endocrinol.* **2015**, *2015*, 423717.
54. O'Dea, A.; Tierney, M.; McGuire, B.E.; Newell, J.; Glynn, L.G.; Gibson, I.; Noctor, E.; Danyliv, A.; Connolly, S.B,; Dunne, F.P. Can the onset of type 2 diabetes be delayed by a group-based lifestyle intervention in women with prediabetes following gestational diabetes mellitus (GDM)? Findings from a randomized control mixed methods trial. *J. Diabetes Res.* **2015**, *2015*, 798460.
55. Smith, B.J.; Cinnadaio, N.; Cheung, N.W.; Bauman, A.; Tapsell, L.C.; van der Ploeg, H.P. Investigation of a lifestyle change strategy for high-risk women with a history of gestational diabetes. *Diabetes Res. Clin. Pract.* **2014**, *106*, e60–e63.
56. Potzel, A.L.; Gar, C.; Banning, F.; Sacco, V.; Fritsche, A.; Fritsche, L.; Müssig, K.; Dauben, L.; Seissler, J.; Lechner, A. A novel smartphone app to change risk behaviors of women after gestational diabetes: A randomized controlled trial. *PLoS ONE* **2022**, *17*, e0267258.
57. Brazeau, A.-S.; Leong, A.; Meltzer, S.J.; Cruz, R.; DaCosta, D.; Hendrickson-Nelson, M.; Joseph, L.; Dasgupta K.; MoMM study group. Group-based activities with on-site childcare and online support improve glucose tolerance in women within 5 years of gestational diabetes pregnancy. *Cardiovasc. Diabetol.* **2014**, *13*, 104.
58. Cheung, N.W.; Smith, B.; Van Der Ploeg, H.; Cinnadaio, N.; Bauman, A. A pilot structured behavioural intervention trial to increase physical activity among women with recent gestational diabetes. *Diabetes Res. Clin. Pract.* **2011**, *92*, e27–e29.
59. Cheung, N.W. Post-pregnancy intervention for the prevention of T2 diabetes. *Obstet. Med.* **2022**, *15* (Suppl. 1), 15–16.
60. Ferrara, A.; Hedderson, M.M.; Brown, S.D.; Albright, C.L.; Ehrlich, S.F.; Tsai, A.-L.; Caan, B.J.; Sternfeld, B.; Gordon, N.P.; Schmittdiel, J.A.; et al. The comparative effectiveness of diabetes prevention strategies to reduce postpartum weight retention in women with gestational diabetes mellitus: The Gestational Diabetes' Effects on Moms (GEM) cluster randomized controlled trial. *Diabetes Care* **2016**, *39*, 65–74.
61. Liu, H.; Wang, L.; Zhang, S.; Leng, J.; Li, N.; Li, W.; Wang, J.; Tian, H.; Qi, L.; Yang, X.; et al. One-year weight losses in the Tianjin Gestational Diabetes Mellitus Prevention Programme: A randomized clinical trial. *Diabetes Obes. Metab.* **2018**, *20*, 1246–1255.
62. Kapoor, D.; Gupta, Y.; Desai, A.; Praveen, D.; Joshi, R.; Rozati, R.; Bhatla, N.; Prabhakaran, D.; Reddy, P.; Patel, A. et al. Lifestyle intervention programme for Indian women with history of gestational diabetes mellitus. *Glob. Health Epidemiol. Genom.* **2019**, *4*, e1.

63. Kim, C.; Draska, M.; Hess, M.; Wilson, E.; Richardson, C. A web-based pedometer programme in women with a recent history of gestational diabetes. *Diabet. Med.* **2012**, *29*, 278–283.
64. Kim, S.-H.; Kim, H.J.; Shin, G. Self-management mobile virtual reality program for women with gestational diabetes. *Int. J. Environ. Res. Public Health* **2021**, *18*, 1539.
65. Lee, K.W.; Tan, S.F.; Omar, A.; Nasir, N.H.; Ching, S.M.; Mohd Noor, M.K.; Masdor, N.A.; Lee, Y.S.; Noordin, S.; Mohamed, H. Effectiveness of system-based intervention in reducing incidence of type 2 diabetes and to improve the postnatal metabolic profiles in women with gestational diabetes mellitus: A randomized controlled study. *Gynecol. Endocrinol.* **2022**, *38*, 55–62.
66. Aroda, V.; Christophi, C.; Edelstein, S.; Zhang, P.; Herman, W.; Barrett-Connor, E.; Delahanty, L.M.; Montez, M.G.; Ackermann, R.T.; Zhuo, X.; et al. The effect of lifestyle intervention and metformin on preventing or delaying diabetes among women with and without gestational diabetes: The Diabetes Prevention Program outcomes study 10-year follow-up. *J. Clin. Endocrinol. Metab.* **2015**, *100*, 1646–1653.
67. Ratner, R.E.; Christophi, C.A.; Metzger, B.E.; Dabelea, D.; Bennett, P.H.; Pi-Sunyer, X.; Fowler, S.; Kahn, S.E.; Diabetes Prevention Program Research Group. Prevention of diabetes in women with a history of gestational diabetes: Effects of metformin and lifestyle interventions. *J. Clin. Endocrinol. Metab.* **2008**, *93*, 4774–4779.
68. McIntyre, H.D.; Peacock, A.; Miller, Y.D.; Koh, D.; Marshall, A.L. Pilot study of an individualised early postpartum intervention to increase physical activity in women with previous gestational diabetes. *Int. J. Endocrinol.* **2012**, *2012*, 892019.
69. Barton, K.I.; Donovan, L.; Giroux, I.; Miller, D.; Mottola, M.; McManus, R. Glycated hemoglobin measurements at three, 12 and 24 months postpartum after gestational diabetes. *Clin. Investig. Med.* **2019**, *42*, E37.
70. Nicholson, W.K.; Beckham, A.J.; Hatley, K.; Diamond, M.; Johnson, L.-S.; Green, S.L.; Tate, D. The Gestational Diabetes Management System (GooDMomS): Development, feasibility and lessons learned from a patient-informed, web-based pregnancy and postpartum lifestyle intervention. *BMC Pregnancy Childbirth* **2016**, *16*, 277.
71. O'Reilly, S.; Versace, V.; Mohebbi, M.; Lim, S.; Janus, E.; Dunbar, J. The effect of a diabetes prevention program on dietary quality in women with previous gestational diabetes. *BMC Women's Health* **2019**, *19*, 88.
72. Pérez-Ferre, N.; Del Valle, L.; Torrejón, M.J.; Barca, I.; Calvo, M.I.; Matía, P.; Rubio, M.A.; Calle-Pascual, A.L. Diabetes mellitus and abnormal glucose tolerance development after gestational diabetes: A three-year, prospective, randomized, clinical-based, Mediterranean lifestyle interventional study with parallel groups. *Clin. Nutr.* **2015**, *34*, 579–585.
73. Rautio, N.; Jokelainen, J.; Korpi-Hyövälti, E.; Oksa, H.; Saaristo, T.; Peltonen, M.; Moilanen, L.; Vanhala, M.; Uusitupa,M.; Tuomilehto,J.; et al. Lifestyle intervention in prevention of type 2 diabetes in women with a history of gestational diabetes mellitus: One-year results of the FIN-D2D project. *J. Women's Health* **2014**, *23*, 506–512.
74. Shek, N.W.M.; Ngai, C.S.W.; Lee, C.P.; Chan, J.Y.C.; Lao, T.T.H. Lifestyle modifications in the development of diabetes mellitus and metabolic syndrome in Chinese women who had gestational diabetes mellitus: A randomized interventional trial. *Arch. Gynecol. Obstet.* **2014**, *289*, 319–327.
75. Yu, S.; Wen, J.W.; Miao, L.H.; Hua, H.G. Effects of lifestyle intervention on the weight of postpartum women with gestational diabetes mellitus and impaired glucose tolerance. *J. Nurs. Adm.* **2012**, *12*, 772.
76. Shyam, S.; Arshad, F.; Abdul Ghani, R.; Wahab, N.A.; Safii, N.S.; Nisak, M.Y.B.; Chinna, K.; Kamaruddin, N.A. Low glycaemic index diets improve glucose tolerance and body weight in women with previous history of gestational diabetes: A six months randomized trial. *Nutr. J.* **2013**, *12*, 68.
77. Shyam, S.; Fatimah, A.; Rohana, A.; Norasyikin, A.; Nik Shanita, S.; Chinna, K.; Mohod-Yusof B.N.; Nor, A.K. Effect of including glycaemic index (GI) nutrition education, within the conventional healthy dietary recommendation framework, on body weight and composition of women with prior gestational diabetes mellitus: Results from a one-year randomised controlled trial. *J. Nutr.* **2015**, *21*, 269–283.
78. Wein, P.; Beischer, N.; Harris, C.; Permezel, M. A trial of simple versus intensified dietary modification for prevention of progression to diabetes mellitus in women with impaired glucose tolerance. *Aust. N. Z. J. Obstet. Gynaecol.* **1999**, *39*, 162–166.
79. Yu, X.; Wu, X.; Zhang, Y.; Mao, Y. Effects of lifestyle intervention on insulin resistance and islet B-cell function in postpartum gestational diabetes patients with impaired glucose regulation. *Chin. J. Prev. Control. Chronic* **2012**, *20*, 560–562.
80. Zilberman-Kravits, D.; Meyerstein, N.; Abu-Rabia, Y.; Wiznitzer, A.; Harman-Boehm, I. The impact of a cultural lifestyle intervention on metabolic parameters after gestational diabetes mellitus a randomized controlled trial. *Matern. Child Health J.* **2018**, *22*, 803–811.

81. Yuen, L.; Saeedi, P.; Riaz, M.; Karuranga, S.; Divakar, H.; Levitt, N.; Yang, X.; Simmons, D. Projections of the prevalence of hyperglycaemia in pregnancy in 2019 and beyond: Results from the International Diabetes Federation Diabetes Atlas. *Diabetes Res. Clin. Pract.* **2019**, *157*, 107841.
82. Hamman, R.F.; Wing, R.R.; Edelstein, S.L.; Lachin, J.M.; Bray, G.A.; Delahanty, L.; Hoskin, M.; Kriska, A.M.; Mayer-Davis, E.J.; Pi-Sunyer, X.; et al. Effect of weight loss with lifestyle intervention on risk of diabetes. *Diabetes Care* **2006**, *29*, 2102–2107.
83. Carter-Edwards, L.; Østbye, T.; Bastian, L.A.; Yarnall, K.S.; Krause, K.M.; Simmons, T.-J. Barriers to adopting a healthy lifestyle: Insight from postpartum women. *BMC Res. Notes* **2009**, *2*, 161.
84. Saligheh, M.; McNamara, B.; Rooney, R. Perceived barriers and enablers of physical activity in postpartum women: A qualitative approach. *BMC Pregnancy Childbirth* **2016**, *16*, 131.
85. Al-Dhahir, I.; Reijnders, T.; Faber, J.S.; van den Berg-Emons, R.J.; Janssen, V.R.; Kraaijenhagen, R.A.; Valentijn T Visch, V.T.; Chavannes, N.H.; Evers, A.W. The barriers and facilitators of eHealth-based lifestyle intervention programs for people with a low socioeconomic status: Scoping review. *J. Med. Internet Res.* **2022**, *24*, e34229.
86. Fontil, V.; McDermott, K.; Tieu, L.; Rios, C.; Gibson, E.; Sweet, C.C.; Payne, M.; Lyles, C.R. Adaptation and feasibility study of a digital health program to prevent diabetes among low-income patients: Results from a partnership between a digital health company and an academic research team. *J. Diabetes Res.* **2016**, *2016*, 8472391.
87. Whitehead, L.; Talevski, J.; Fatehi, F.; Beauchamp, A. Barriers to and Facilitators of Digital Health Among Culturally and Linguistically Diverse Populations: Qualitative Systematic Review. *J. Med. Internet Res.* **2023**, *25*, e42719.
88. Joiner, K.L.; Nam, S.; Whittemore, R. Lifestyle interventions based on the diabetes prevention program delivered via eHealth: A systematic review and meta-analysis. *Prev. Med.* **2017**, *100*, 194–207.
89. Dugani, S.B.; Mielke, M.M.; Vella, A. Burden and management of type 2 diabetes in rural United States. *Diabetes/Metab. Res. Rev.* **2021**, *37*, e3410.
90. AIHW. Diabetes: Australian Facts. Available online: https://www.aihw.gov.au/reports/diabetes/diabetes/contents/how-common-is-diabetes/all-diabetes (accessed on 9 February 2023).
91. Harding, C.; Seal, A.; Mills, N. Evaluation of a Lifestyle and Weight Management Program Within Rural General Practice. *J. Prim. Care Community Health* **2022**, *13*, 21501319221084166.
92. Park, J.H.; Moon, J.H.; Kim, H.J.; Kong, M.H.; Oh, Y.H. Sedentary lifestyle: Overview of updated evidence of potential health risks. *Korean J. Fam. Med.* **2020**, *41*, 365.
93. Mbuagbaw, L.; Aves, T.; Shea, B.; Jull, J.; Welch, V.; Taljaard, M. Yoganathan, M.; Greer-Smith, R.; Wells, G.; Tugwell, P. Considerations and guidance in designing equity-relevant clinical trials. *Int. J. Equity Health* **2017**, *16*, 93.
94. Kauh, T.J.; Minnis, T.A.; Anand, M.; Berry, M.; Gold, R. Building an Equitable Future Through Data Disaggregation. *Health Equity* **2023**, *7*, 251–260.
95. Yang, J.; Xia, Y.; Sun, Y.; Guo, Y.; Shi, Z.; do Vale Moreira, N.C.; Zuo, H.; Hussain, A. Effect of lifestyle intervention on HbA1c levels in overweight and obese adults with type 2 diabetes across ethnicities: A systematic review and meta-analysis of randomized controlled trials. *Diabetes Res. Clin. Pract.* **2023**, *199*, 110662.
96. Lynch, E.B.; Mack, L.; Avery, E.; Wang, Y.; Dawar, R.; Richardson, D.; Keim, K.; Ventrelle, J.; Appelhans, B.M.; Tahsin, B.; et al. Randomized trial of a lifestyle intervention for urban low-income African Americans with type 2 diabetes. *J. Gen. Intern. Med.* **2019**, *34*, 1174–1183.
97. Khare, M.M.; Cursio, J.F.; Locklin, C.A.; Bates, N.J.; Loo, R.K. Lifestyle intervention and cardiovascular disease risk reduction in low-income Hispanic immigrant women participating in the Illinois WISEWOMAN program. *J. Community Health* **2014**, *39*, 737–746.
98. Burgess, E.; Hassmén, P.; Pumpa, K.L. Determinants of adherence to lifestyle intervention in adults with obesity: A systematic review. *Clin. Obes.* **2017**, *7*, 123–135.
99. Coupe, N.; Cotterill, S.; Peters, S. Tailoring lifestyle interventions to low socio-economic populations: A qualitative study. *BMC Public Health* **2018**, *18*, 967.
100. Fokas, E. Religion and Human Rights in Greece. In *Global Eastern Orthodoxy*; Springer: Berlin/Heidelberg, Germany, 2020; pp. 101–124.
101. Lim, F.K.; Sng, B.B. Social media, religion and shifting boundaries in globalizing China. *Glob. Media China* **2020**, *5*, 261–274.
102. Bosch-Capblanch, X.; Zuske, M.-K.; Auer, C. Research on subgroups is not research on equity attributes: Evidence from an overview of systematic reviews on vaccination. *Int. J. Equity Health* **2017**, *16*, 95.

103. Koenig, H.G. Religion, spirituality, and health: The research and clinical implications. *Int. Sch. Res. Not.* **2012**, *2012*, 278730.
104. Swihart, D.L.; Yarrarapu, S.N.S.; Martin, R.L. *Cultural Religious Competence in Clinical Practice*; StatPearls Publishing: Treasure Island, FL, USA, 2018.
105. Rivenbark, J.G.; Ichou, M. Discrimination in healthcare as a barrier to care: Experiences of socially disadvantaged populations in France from a nationally representative survey. *BMC Public Health* **2020**, *20*, 31.
106. Tan, S.T.; Low, P.T.A.; Howard, N.; Yi, H. Social capital in the prevention and management of non-communicable diseases among migrants and refugees: A systematic review and meta-ethnography. *BMJ Glob. Health* **2021**, *6*, e006828.
107. Sanjari, M.; Aalaa, M.; Amini, M.R.; Aghaei Meybodi, H.R.; Qorbani, M.; Adibi, H.; et al. Social-capital determinants of the women with diabetes: A population-based study. *J. Diabetes Metab. Disord.* **2021**, *20*, 511–521.
108. Jiang, H.; Xingling, C.; Yuxiu, H.; Feng, T. Progress and Inspiration of Social Capital in the Prevention and Control of Diabetes. *Iran. J. Public Health* **2020**, *49*, 196–197.
109. Keyvanara, M.; Afshari, M.; Dezfoulian, E. The relationship between social capital and quality of life among patients referring to diabetes centers in Isfahan, Iran. *J. Diabetes Res.* **2018**, *2018*, 9353858.
110. Grootaert, C. *Measuring Social Capital: An Integrated Questionnaire*; World Bank Publications: Washington, DC, USA, 2004.

Disclaimer/Publisher's Note: The statements, opinions and data contained in all publications are solely those of the individual author(s) and contributor(s) and not of MDPI and/or the editor(s). MDPI and/or the editor(s) disclaim responsibility for any injury to people or property resulting from any ideas, methods, instructions or products referred to in the content.
